# Mechanistic Study on the Degradation of Hydrolysable Core-Crosslinked Polymeric Micelles

**DOI:** 10.1021/acs.langmuir.3c01399

**Published:** 2023-08-15

**Authors:** Erik R. Hebels, Mies J. van Steenbergen, Ragna Haegebaert, Cornelis W. Seinen, Barbara S. Mesquita, Antoinette van den Dikkenberg, Katrien Remaut, Cristianne J. F. Rijcken, Bas G. P. van Ravensteijn, Wim E. Hennink, Tina Vermonden

**Affiliations:** † Department of Pharmaceutics, Utrecht Institute for Pharmaceutical Sciences (UIPS), Utrecht University, 3508 TB Utrecht, The Netherlands; ‡ Cristal Therapeutics, 6229 EV Maastricht, The Netherlands; § Laboratory for General Biochemistry and Physical Pharmacy, Ghent University, Ottergemsesteenweg 460, 9000 Gent, Belgium; ∥ Division Laboratories, Pharmacy and Biomedical Genetics, Central Diagnostic Lab, University Medical Center Utrecht, Heidelberglaan 100, 3584 CX Utrecht, The Netherlands

## Abstract

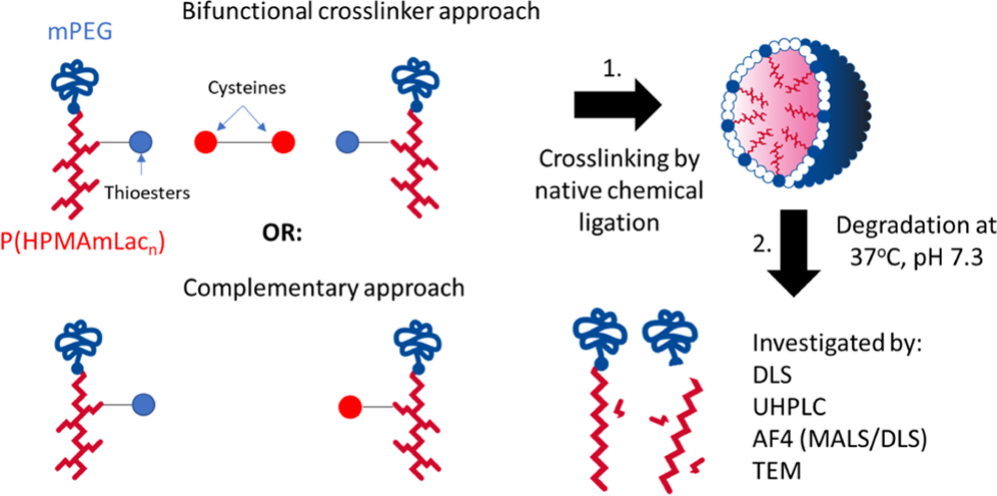

Core-crosslinked polymeric micelles (CCPMs) are an attractive class of nanocarriers for drug delivery. Two crosslinking approaches to form CCPMs exist: either via a low-molecular-weight crosslinking agent to connect homogeneous polymer chains with reactive handles or via cross-reactive handles on polymers to link them to each other (complementary polymers). Previously, CCPMs based on methoxy poly(ethylene glycol)-*b*-poly[*N*-(2-hydroxypropyl) methacrylamide-lactate] (mPEG-*b*-PHPMAmLac*_n_*) modified with thioesters were crosslinked via native chemical ligation (NCL, a reaction between a cysteine residue and thioester resulting in an amide bond) using a bifunctional cysteine containing crosslinker. These CCPMs are degradable under physiological conditions due to hydrolysis of the ester groups present in the crosslinks. The rapid onset of degradation observed previously, as measured by the light scattering intensity, questions the effectiveness of crosslinking via a bifunctional agent. Particularly due to the possibility of intrachain crosslinks that can occur using such a small crosslinker, we investigated the degradation mechanism of CCPMs generated via both approaches using various analytical techniques. CCPMs based on complementary polymers degraded slower at pH 7.4 and 37 °C than CCPMs with a crosslinker (the half-life of the light scattering intensity was approximately 170 h versus 80 h, respectively). Through comparative analysis of the degradation profiles of the two different CCPMs, we conclude that partially ineffective intrachain crosslinks are likely formed using the small crosslinker, which contributed to more rapid CCPM degradation. Overall, this study shows that the type of crosslinking approach can significantly affect degradation kinetics, and this should be taken into consideration when developing new degradable CCPM platforms.

## Introduction

Polymeric micelles (PMs) are sub-100 nm-sized colloidal particles composed of amphiphilic block copolymers, with a hydrophobic block that constitutes the PM core and a hydrophilic block forming the shell in aqueous environments at polymer concentrations above the critical micellization concentration (CMC). PMs are a promising class of nanocarriers for the formulation and delivery of therapeutics in the body.^1−3^ Crosslinking of the PM core by covalent bonds to form core-crosslinked polymeric micelles (CCPMs) greatly enhances the stability of PMs in circulation by halting the equilibrium between unimers and the PM state, which can lead to rapid and unwanted destabilization upon administration.^4−6^

Crosslinking of PMs has been carried out using diverse chemistries including, among others, radical polymerization,^7−10^ copper-catalyzed click chemistry,^11,12^*N*-acryloxysuccinimide with amine coupling,^13^ bis-benzophenone-mediated photo-crosslinking,^14^ Diels–Alder reaction,^15^ disulfide exchange,^16,17^ thiol oxidation,^18^ and native chemical ligation (NCL).^19^ Generally speaking, these crosslinking strategies can be divided into two principal approaches: (1) by using a bifunctional crosslinking agent to connect polymer chains with reactive handles and (2) using cross-reactive handles (referred to in this paper as complementary) present on the different polymer chains to link with one another.^4^ Additionally, polymers and/or crosslinks constituting the CCPMs have most commonly been designed to degrade under physiological conditions by pH-dependent hydrolysis,^8,9,20,21^ reduction-sensitive cleavage^16,22−24^ as well as combinations of these,^12,17,25−27^ and to a lesser extent photosensitive cleavage.^28^ Although these degradation strategies are mostly designed to facilitate triggered cargo release at the target site, the ability of CCPMs to degrade under physiologically relevant conditions is important for the clearance of the nanocarrier following administration *in vivo*.

To facilitate degradation, polymers based on degradable *N*-2-hydroxypropyl methacrylamide-lactate (HPMAmLac*_n_*) monomers have been developed previously. Ester bonds present in the side chains of PHPMAmLac_n_ can be hydrolyzed under physiological conditions (pH 7.4 and 37 °C), which results in hydrophilic HPMA-rich polymer chains and lactic acid that are expected to be cleared from circulation (if PHPMA sizes are less than 100 kDa^29^) and metabolized, respectively.^30−32^ Additionally, polymers based on HPMAmLac_n_ are thermosensitive,^30^ allowing for convenient temperature-induced micellization for PEG containing block copolymers.^31^ By functionalization of the polymer side chains with reactive handles, crosslinking of the micellar core after temperature-induced micellization can be achieved as was done via free radical reactions of methacrylated methoxy poly(ethylene glycol)-*b*-poly[*N*-(2-hydroxypropyl) methacrylamide-lactate] (mPEG-*b*-PHPMAmLac*_n_*) block copolymers, also known as CriPec (which has been evaluated as a drug carrier in (pre)clinical studies).^10,33^

**Scheme 1:**
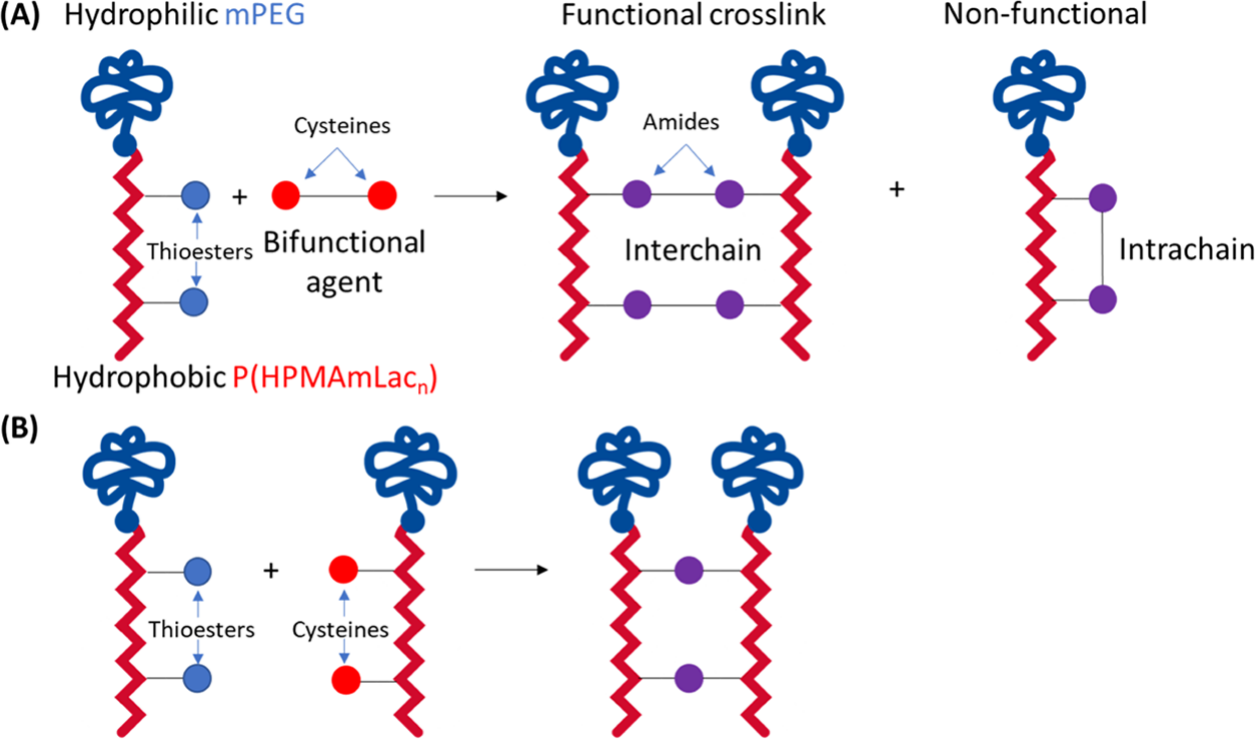
Representation of Possible Linkage Outcomes Using (A) a Bifunctional Crosslinking Agent Approach or (B) a Complementary Polymer Approach

Recently, we reported the use of NCL (a reaction between a cysteine and a thioester forming an amide bond) as an orthogonal crosslinking reaction in mPEG-*b*-PHPMAmLac*_n_* block copolymers for the formation of CCPM using a bifunctional crosslinking agent.^34^ The advantage of this NCL crosslinking strategy is that no free radical reactions are needed that can potentially damage sensitive cargo. Briefly, by increasing the temperature of an aqueous solution of thermosensitive mPEG-*b*-PHPMAmLac*_n_* block copolymers modified with thioesters, micelles were formed, which were subsequently stabilized by a crosslinker with two cysteine handles, allowing amide bond formation via NCL. Degradation of these CCPMs under physiological conditions occurs through ester hydrolysis of the HPMAmLac_n_ side chains present in the crosslinks, analogous to the CriPec system. Although stable CCPMs (resistant to destabilization by surfactants) with tunable sizes were achieved by this approach, a rapid onset of degradation under physiological conditions, as measured by the decreasing light scattering intensity, was observed. This rapid onset of degradation was unexpected as it suggests that the CCPMs start to disintegrate immediately upon exposure to these conditions, without a lag time, which would be expected from hydrolysis of the excessive number of crosslinks (∼6 connection points per polymer chain were present^34^) before destabilization of the CCPMs. A possible explanation is that the use of a bifunctional crosslinking agent to prepare CCPMs can lead to intramolecular and thus ineffective crosslinks (see Scheme 1A).

In the present study, the degradation of CCPMs formed via NCL with a bifunctional crosslinker was investigated in depth. The degradation of these CCPMs was compared with CCPMs formed from complementary polymers by interchain crosslinking, where intrachain crosslinking cannot occur (Scheme 1B). Several analytical techniques were employed to gain insights into the degradation mechanism of both types of micelles, including dynamic light scattering (DLS), ultra-high-performance liquid chromatography (UHPLC), asymmetric flow field flow fractionation (AF4), multiangle light scattering (MALS), and transmission electron microscopy (TEM) imaging.

## Experimental Section

### Materials

All materials were obtained from Sigma-Aldrich (Zwijndrecht, The Netherlands) unless indicated otherwise. Ethyl thioglycolate-succinic acid (ETSA) was synthesized according to a previously published procedure.^35^ The 2-(methoxy polyethylene glycol)-4,4-azobis(4-cyanopentanoic acid) (mPEG_5000_)_2_-ABCPA free radical macroinitiator was synthesized according to a previously published procedure.^36^*N*-2-Hydroxypropyl methacrylamide monolactate (HPMAmLac_1_) and dilactate (HPMAmLac_2_) were provided by Cristal Therapeutics (the syntheses have been described previously).^37^ All solvents were obtained from Biosolve (Valkenswaard, The Netherlands).

### Synthesis

#### Bifunctional Crosslinker Synthesis (Compound **2**)

**Scheme 2:**
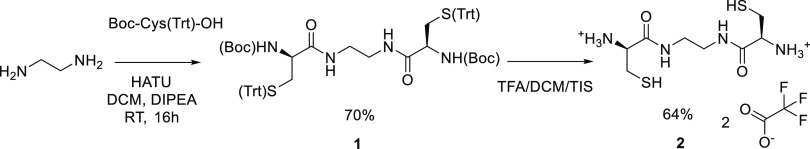
Synthesis of the *N*,*N*′-Bis-cysteinyl-ethylendiamine Crosslinker (Compound **2**)

A crosslinker with two cysteine residues was synthesized, similar to a previously published procedure.^38^ Boc-Cys(Trt)-OH (1.46 g, 3.15 mmol) and 1-[bis(dimethylamino)methylene]-1*H*-1,2,3-triazolo[4,5-*b*]pyridinium 3-oxid hexafluorophosphate (HATU) (1.14 g, 3.00 mmol) were dissolved in 10 mL of dry dichloromethane (DCM), followed by addition of *N*,*N*-diisopropylethylamine (DIPEA) (1.57 mL, 8.99 mmol) and finally ethylene diamine (0.10 mL, 1.50 mmol). After overnight stirring at RT, the reaction mixture was diluted with 30 mL of DCM, washed 3 times with 30 mL of saturated aqueous NaHCO_3_ solution, dried over sodium sulfate, and concentrated. Purification was done via silica chromatography using ethyl acetate/hexane 7:3 as the eluent (*R*_f_ = 0.5), which after concentrating yielded 0.99 g (70%) of *N*,*N*′-bis[*N*-*tert*-butyloxycarbonyl-S-triphenylmethyl-cysteinyl] ethylendiamine (Scheme 2, compound **1**), an off-white gooey semisolid (see Figure S1.1 for NMR). ^1^H NMR (400 MHz, DMSO): δ 7.85 (s, 2H), 7.37 – 7.20 (m, 30H), 6.84 (d, *J* = 8.5 Hz, 2H), 3.89 (q, *J* = 7.5 Hz, 2H), 3.06–2.95 (m, 4H), 2.33 (qd, *J* = 11.9, 6.9 Hz, 4H), 1.37 (s, 18H).

##### Deprotection

To remove the tert-butyloxycarbonyl (Boc) and trityl (Trt) protecting groups, compound **1** (0.30 g, 0.32 mmol) was added to 10 mL of a DCM/trifluoroacetic acid (TFA)/triisopropyl silane (TIS) solution (50:47:3% volume), and the mixture was subsequently stirred for 15 min, after which the formed *N*,*N*′-bis-cysteinyl-ethylendiamine (Scheme 2, compound **2**) was precipitated in diethyl ether. After rinsing and centrifuging with additional diethyl ether, the precipitate was dried under a N_2_ flow. The obtained white solid was then dissolved in 5 mL of milliQ water and purified by preparative reverse-phase high-performance liquid chromatography (Prep-RP-HPLC) on a Waters 2535 quaternary gradient module with a Waters 2489 UV–visible detector (detection at 210 and 280 nm) and a ReproSil-Pur 120 C18-AQ (10 μm, 25 mm × 250 mm, Dr. Maisch) column. Acetonitrile (ACN)/water supplemented with 0.1% formic acid was used as the eluent at a flow of 25 mL/min and a gradient of 5–100% ACN over 60 min. After freeze-drying, the pure fractions yielded 73 mg (64%) of fluffy white solid (see Figure S1.2 for NMR). ^1^H NMR (400 MHz, DMSO): δ 8.60–8.51 (m, 2H), 3.90 (t, *J* = 5.6 Hz, 2H), 3.22–3.14 (m, 2H), 2.92 (dd, *J* = 5.6, 1.8 Hz, 4H). As no formate counterion protons were detected, ^19^F NMR was conducted to quantify the amount of trifluoroacetate counterions remaining using trifluoroethanol as the internal standard, described in the Nuclear Magnetic Resonance (NMR) Spectroscopy section below.

#### Thermosensitive Block Copolymer Synthesis and Its Derivatization with Thioester or Cysteine Groups

**Scheme 3:**
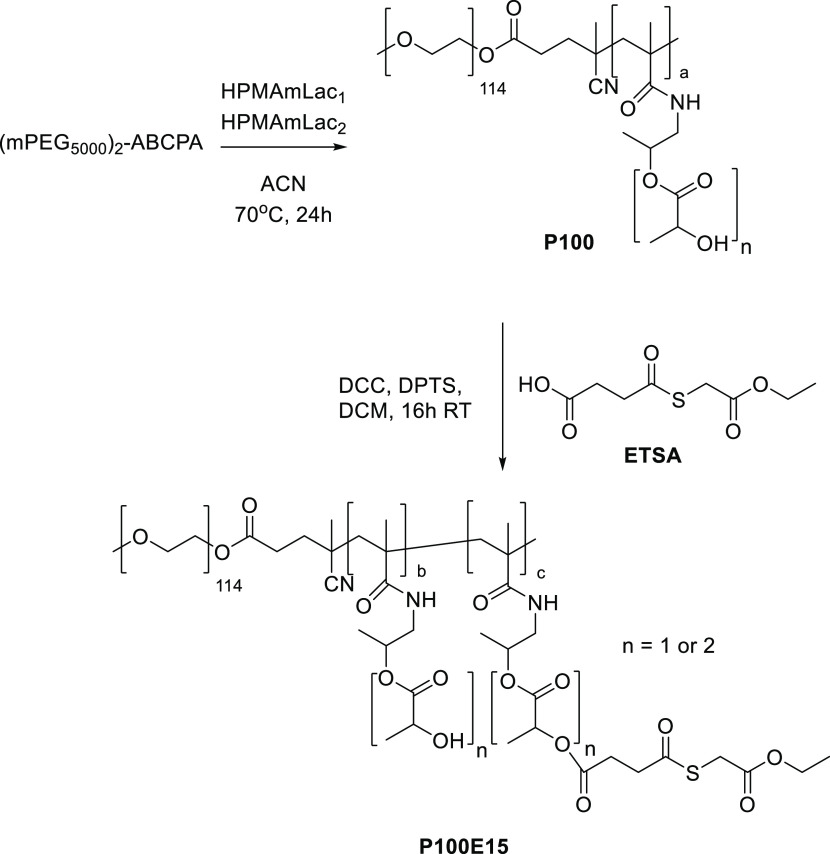
Synthesis of mPEG_5000_-*b*-P(HPMAmLac_1_-*co*-HPMAmLac_2_) (Polymer **P100**) by Free Radical Polymerization Using a (Methoxy polyethylene glycol)_2_-4,4-azobis(4-cyanopentanoic acid) ((mPEG_5000_)_2_-ABCPA) Macroinitiator and Subsequent ETSA Coupling to Obtain the Polymer **P100E15**

A block copolymer poly(ethylene glycol)-*b*-poly(*N*-(2-hydroxypropyl) methacrylamide-lactate) (mPEG_5000_-*b*-P(HPMAmLac*_n_*-*co*-HPMAmLac*_n_*), further abbreviated as **P100**) was synthesized by free radical polymerization following a previously published procedure (Scheme 3).^10,34^ In short, the (mPEG_5000_)_2_-ABCPA macroinitiator (400 mg) was weighed into a Schlenk tube followed by HPMAm-monolactate (HPMAmLac_1_, 457 mg) and HPMAm-dilactate (HPMAmLac_2_, 544 mg, 1.97 mL from a 276 mg/mL stock solution in ACN), resulting in a monomer/initiator molar ratio of 100/1 and an HPMAmLac_1_/HPMAmLac_2_ molar feed ratio of 53:47. Additional ACN (2.7 mL) was added to dilute the mixture to a final monomer (with initiator) concentration of 300 mg/mL. The tube was sealed by a rubber septum, and five freeze–pump–thaw cycles were applied, backflushed with nitrogen, and placed into an oil bath of 70 °C for 24 h. Next, the reaction mixture was cooled down to RT, and the obtained polymer was precipitated 3 times in diethyl ether and dried under nitrogen. The polymer was then dissolved in 1:1 ACN/water and freeze-dried. The obtained block copolymer **P100**, yielding 1.06 g (76%), was analyzed by NMR and GPC (Figures S1.3 and S2.1).

##### Thioester Derivatization

**P100** was functionalized with ETSA to obtain the mPEG_5000_-*b*-P(HPMAmLac*_n_*-*co*-HPMAmLac*_n_*-ETSA) block copolymer (Scheme 3, abbreviated as **P100E15**). Stock solutions of ETSA (50 mg/mL), *N*,*N*-dimethylaminopyridinium *p*-toluenesulfonate (DPTS, 20 mg/mL), and *N*,*N*′*-*dicyclohexylcarbodiimide (DCC, 50 mg/mL) in dry DCM were prepared. **P100** (16 kDa, determined by NMR) was weighed (1050 mg, 66 μmol) and dissolved in dry DCM (final concentration of 100 mg/mL), followed by addition of ETSA (97 mg, 440 μmol), with a feed ratio of 15 mol % relative to HPMAmLac*_n_* functionalities, DPTS (12 mg, 44 μmol) and DCC (100 mg, 482 μmol). The reaction mixture was stirred at RT for 24 h. The mixture was then filtered using a 0.2 μm PTFE syringe filter to remove precipitated *N*,*N*′-dicyclohexylurea (DCU). Subsequently, the polymer was precipitated twice in diethyl ether and dried under vacuum overnight. The obtained block copolymer **P100E15**, yielding 0.90 g (86%), was analyzed by NMR and GPC (Figures S1.4 and S2.1).

##### Cysteine Derivatization

**Scheme 4:**
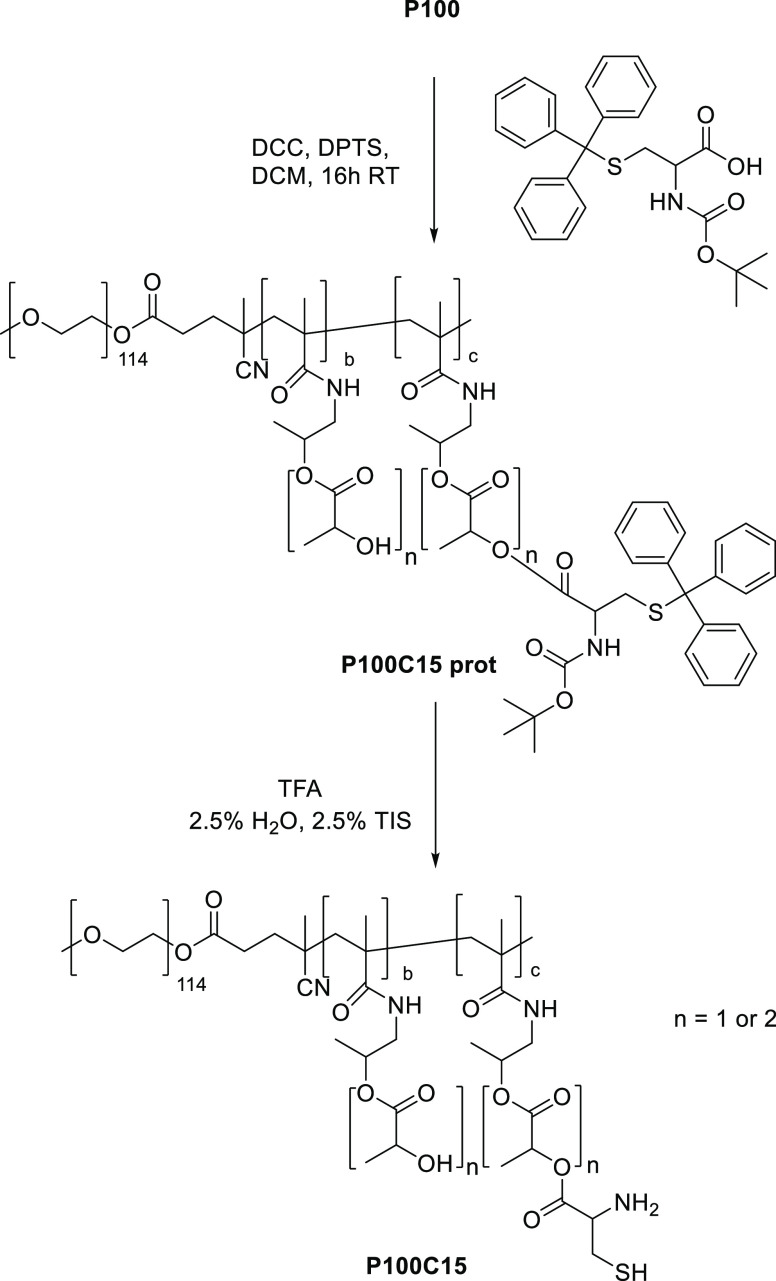
Synthesis of the Cysteine-Modified Polymer **P100C15**

The same procedure was applied as described above, with Boc-Cys(Trt)-OH (50 mg/mL in dry DCM) instead of ETSA, resulting in mPEG_5000_-*b*-P(HPMAmLac*_n_*-*co*-HPMAmLac*_n_*-Cys(Trt)Boc) (Scheme 4, further abbreviated as **P100C15 prot**). **P100C15 prot** was analyzed by NMR spectroscopy using the integral of the Boc protons for the quantification of cysteine moieties (Figure S1.5). Next, Boc and trityl deprotection was carried out by dissolving 300 mg of **P100C15 prot** in 5 mL of DCM followed by addition of 5 mL of a TFA/water/TIS solution (90:5:5%volume) and stirring at RT. After 2 h, the deprotected polymer mPEG_5000_-*b*-P(HPMAmLac*_n_*-*co*-HPMAmLac*_n_*-Cys) (further abbreviated as **P100C15**) was precipitated in diethyl ether and dried under vacuum. **P100C15**, yielding 0.23 g (76%), was analyzed by GPC (Figure S2.1).

#### Core-Crosslinked Polymeric Micelle (CCPM) Formation and Purification

##### Crosslinker Approach

**P100E15** (23 kDa, determined by NMR, 120 mg, 5.2 μmol of polymer chains, 46.0 μmol of ETSA handles) was dissolved in 6 mL of phosphate buffer (100 mM Na_2_HPO_4_, adjusted to pH 7.4 using HCl) while stirring in an ice bath. The *N*,*N*′-bis-cysteinyl-ethylendiamine crosslinker compound **2** (9.3 mg of the TFA salt (see Figure S1.2), 18.8 μmol containing 37.6 μmol of cysteine handles (0.80 ± 0.05 equiv of cysteine to ETSA handles)) was dissolved (75 mg/mL) in milliQ, and tris(2-carboxyethyl)phosphine (TCEP, 19.5 mg, 78.0 μmol) in milliQ (112.5 mg/mL) was added to reduce possible present disulfide bonds. After 20 min, the crosslinker/TCEP mixture was placed in a water bath of 37 °C while stirring, and the polymer solution was then added. After 1.5 h, the dispersion of formed CCPMs was filtered through a 0.2 μm RC syringe filter and purified by tangential flow filtration (TFF) against phosphate buffer (100 mM Na_2_HPO_4_, adjusted to pH 7.4 using HCl) employing an mPES membrane (50 kDa, 20 cm^2^) for 40–50 washing volumes. The CCPM dispersion was then filtered again using a 0.2 μm RC syringe filter. The polymer concentration was determined similarly to a previously described total hydrolysis method,^34^ described in the characterization section.

##### Complementary Approach

**P100E15** (60 mg, 2.7 μmol of polymer chains, 23.5 μmol of ETSA handles) and **P100C15** (60 mg, 3.6 μmol of polymer chains, 22.4 μmol of cysteine handles) were separately dissolved in phosphate buffer (100 mM Na_2_HPO_4_, adjusted to pH 7.4 using HCl) to 20 mg/mL while stirring in an ice bath. Then, TCEP (9.5 mg, 38 μmol) was added to **P100C15** to reduce any potential disulfide formation, followed by **P100E15**, and the mixture was heated to 37 °C while stirring (0.95 ± 0.05 equiv of cysteine to ETSA handles). After 1.5 h, the same purification procedure as described for CCPM formation via the small-molecule crosslinker approach was employed.

### Crosslinker, Polymer, and Particle Characterization

#### Nuclear Magnetic Resonance (NMR) Spectroscopy

^1^H and ^19^F NMR spectra were recorded on an Agilent 400-MR NMR spectrometer (Agilent Technologies, Santa Clara). The residual solvent peak of *d*_6_ DMSO (δ = 2.50 ppm) was used to calibrate ^1^H chemical shifts.

#### Trifluoroacetate (TF-Acetate) Quantification in Crosslinker (Compound **2**)

The crosslinker (compound **2**) was weighed (6.0 mg) together with an internal standard, trifluoroethanol (7.1 mg), dissolved in 0.5 mL of *d*_6_ DMSO, and an ^19^F NMR spectrum (Figure S1.6) was recorded with a relaxation delay of 40 s (5-fold exceeding the highest *T*_1_ measured of 6.5 s). Relative integral area was used to calculate the mass of TF-acetate present in the crosslinker sample, and it was found to be 3.0 mg. The expected mass content of two TF-acetate counterions to the two amines in the 6 mg crosslinker sample is 2.8 mg, which is in good agreement with the measured content. The molecular weight of the crosslinker employed in this work is therefore 494.4 g/mol (compound **2** with two TF-acetate ions).

#### Gel Permeation Chromatography (GPC)

GPC analysis was performed using an Alliance 2695 (Waters) chromatography system with two PLgel 5 μm mixed-D columns (Polymer Laboratories) in series at a column temperature of 65 °C and employing a differential refractive index detector. DMF supplemented with 10 mM LiCl was employed as the eluent with an elution rate of 1 mL/min. Sample concentrations were 10 mg/mL with 50 μL injections, and PEGs of narrow and defined molecular weights obtained from PSS (Germany) were used as calibration standards. Recording of data was done with Waters Empower 32 software.

#### Cloud Point (CP) Measurement

The CPs of **P100E15** and **P100C15** in phosphate buffer (100 mM Na_2_HPO_4_, adjusted to pH 7.4 using HCl, 5 mg/mL polymer) were determined by measurement of light scattering at a 90° angle upon the onset of opalescence. Scattered light intensity was measured using a Jasco FP-8300 spectrophotometer at a wavelength of 550 nm. The temperature was ramped from 2 to 50 °C at a rate of 1 °C/min.

#### CCPM Hydrolytic Degradation

Purified CCPMs obtained via the crosslinker and the complementary polymer approach were incubated at 37 °C, and degradation was monitored using the following described techniques over a period of 192 h.

#### Dynamic Light Scattering (DLS)

The hydrodynamic size, scattering intensity, and dispersity of the CCPMs (approximately 15 mg/mL polymer concentration) were determined by DLS using a Malvern Zetasizer nano series S (Malvern Panalytical Ltd., U.K.) with a measurement angle of 173° at a temperature of 37 °C.

For the bulk DLS degradation data, the derived count rate relative to the time point 0 measurement was normalized for the sake of comparison between the two systems (originally 11 and 13 Mcps for the crosslinker and complementary-based CCPMs, respectively, at time point 0 h).

#### Ultra-High-Performance Liquid Chromatography (UHPLC)

UHPLC analysis was performed using an Acquity (Waters, US) chromatography system with an HSS T3 column (1.8 μm, 2.1 × 100 mm^2^, Waters) at a column temperature of 50 °C and employing a Waters TUV detector at 210 nm. KH_2_PO_4_ buffer (10 mM, pH = 2.5) was used as the isocratic eluent at a flow rate of 0.5 mL/min for 2.5 min followed by an increasing gradient of acetonitrile supplemented with 0.1% phosphoric acid from 0 to 90% over 1 min. The injection volume was 5 μL.

Ethyl thioglycolate (ET) formation kinetics of both CCPM systems were determined by repeated injections during crosslinking within the sample holder at 37 °C, employing the same molar ratios described in the Synthesis section above for a 1 mL (instead of 6 mL) sample. ET dilutions (200–1000 μg/mL) in phosphate buffer (100 mM Na_2_HPO_4_, adjusted to pH 7.4 using HCl) were used as reference standards. The ET formation % was calculated from the amount of ET expected based on the cysteine residues added (the cysteine residues being the limiting reagents).

The total polymer content of CCPMs after purification was determined through lactic acid concentration determination by UHPLC after hydrolysis, similarly as reported previously.^39^ Briefly, the micellar dispersion was diluted 5-fold (to a theoretical maximum of ∼4 mg/mL based on polymer feed) in phosphate buffer (100 mM Na_2_HPO_4_, adjusted to pH 7.4 using HCl), 20 μL of the dilution was mixed with 10 μL of NaOH (1 M), and subsequently incubated for 24 h at RT followed by the addition of 20 μL of HCl (1 M). Sodium lactate was employed as the reference standard to determine the lactic acid concentration. The total polymer concentration was calculated as follows: Amount of polymer = measured amount of lactic acid × (*M* + 5000)/[90.08 × (*m* + 2*n*)], where *M* is the *M*_n_ of the thermosensitive block P(HPMAmLac*_n_*) and *m* and *n* are the numbers of repeat units of HPMAmLac_1_ and HPMAmLac_2_ in the block copolymer, respectively (determined by ^1^H NMR).

Lactic acid formation of the degrading CCPMs was quantified by injecting 20 μL of micellar dispersion (∼20 mg/mL theoretical maximum polymer concentration based on feed) diluted with 10 μL of 1 M HCl and 20 μL of milliQ. The intact CCPM peak signals were simultaneously recorded.

#### Asymmetric Flow Field Flow Fractionation (AF4)

AF4 was performed using an AF2000 system (Postnova Analytics GmbH, Germany), equipped with an absorbance 2487 and fluorescence 2475 detector (Waters), a PN3150 RI detector (Postnova Analytics GmbH, Germany), a PN3621b multiangle light scattering (MALS) detector with 21 detection angles with a 488 nm laser (Postnova Analytics GmbH, Germany), and a Zetasizer Nano SZ (Malvern Panalytical Ltd., U.K.). The separation channel included a 500 μm spacer and a regenerated cellulose membrane with a 10 kDa cutoff (Postnova Analytics GmbH, Germany). Phosphate-buffered saline (PBS, 137 mM NaCl, 2.7 mM KCl, 8 mM Na_2_HPO_4_, and 2 mM KH_2_PO_4_, pH 7.4) filtered with an Omipore 0.1 μm PTFE membrane (Merck Millipore Ltd, Ireland) was used as the mobile phase. Samples of CCPMs (60 μL of ∼15 mg/mL) were injected into the channel with an autosampler, focused for 7 min at a focus flow rate of 4.3 mL/min and crossflow of 4.0 mL/min, and separated using the elution profile given in Table 1. Data were analyzed and processed using NovaFFF AF2000 software (Postnova Analytics GmbH, Germany). A sphere model was employed for fitting MALS data and to calculate the radius of gyration (*R*_g_).

**Table 1:** Elution Profile Employed for the Fractionation of CCPMs

elution step	time (min)	crossflow (mL/min)	type	exponent
1	5	4.0	constant	
2	30	4.0–0.10	power	0.2
3	30	0.10–0.05	power	0.8
4	20	0.05–0.00	constant	
5	10	0.00	constant	

#### Transmission Electron Microscopy (TEM) Visualization

For the TEM visualization, 15 μL of CCPM dispersion (1250× diluted in milliQ water) was dropped onto a layer of activated carbon film supported on a 100-mesh hexagonal copper grid and incubated for 15 min. CCPMs/polymers not bound to the activated carbon film were washed away with several drops of PBS pH 7.4. Subsequently, the sample was fixated using 1% glutaraldehyde in PBS for 10 min and the grid was extensively washed with several drops of milliQ water. Next, 150 μL of a negative staining solution (a mixture of 2% uranyl oxalate and 0.15% methylcellulose in an ammonium acetate buffer pH 7.0) was applied for a 15 min incubation, after which the excess was blotted away using a filter paper and the sample was air-dried for at least 1 h at RT. Images were obtained on an FEI Tecnai G2 20 TWIN electron microscope, which was operated at an acceleration voltage of 120 kV and a spot size of 3. Images were recorded using RADIUS software.

## Results and Discussion

**Scheme 5:**
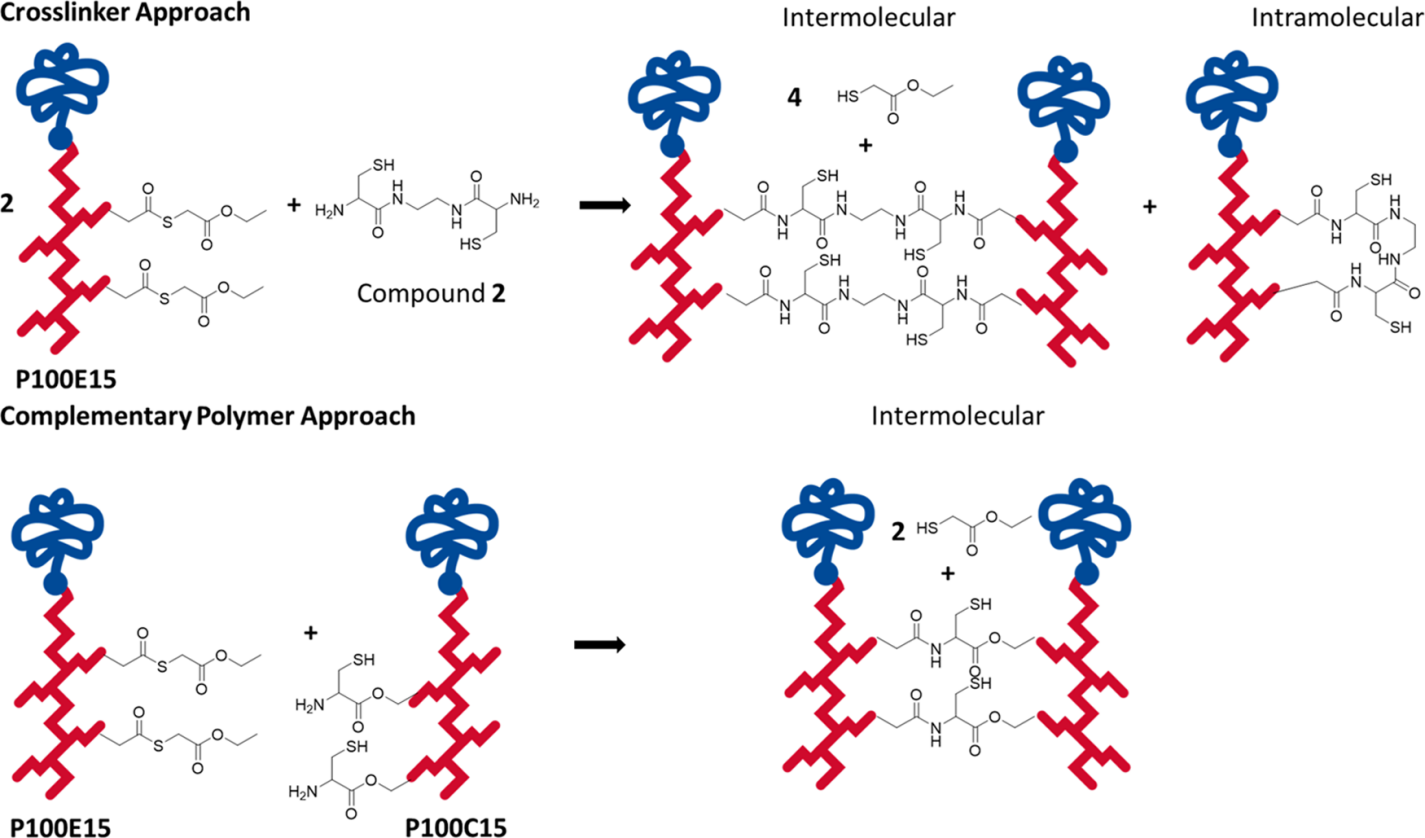
Representation of Two Copolymer Chains Crosslinking via NCL through Either a Crosslinker or a Complementary Polymer Approach Note: **P100E15** has 8.8 thioester units and **P100C15** has 6.3 cysteine units per polymer chain. The crosslinker approach requires two amide bond formations per crosslink, while the complementary approach requires only one.

Native chemical ligation (NCL) is a strategy to prepare CCPMs,^34^ and two crosslinking approaches were evaluated in view of the degradation characteristics of the resulting CCPMs in this study. CCPMs were prepared by crosslinking **P100E15** with a bifunctional agent (compound **2**) or by reacting **P100E15** with **P100C15** (see Scheme 5). The “crosslinker approach” could result in CCPMs with both intra- and intermolecular crosslinks, whereas the “complementary polymer approach” yields CCPMs with only intermolecular crosslinks.

### Crosslinker Synthesis

The bifunctional cysteine crosslinker (compound **2**) was synthesized using an adapted procedure,^38^ and its identity was confirmed by NMR analysis (see Figure S1.2).

### Polymer Synthesis

The synthesized mPEG_5000_-*b*-P(HPMAmLac_1_-*co*-HPMAmLac_2_) (**P100**) polymers had *M*_n_ values of 15.8 and 17.8 kDa (from two different batches) as determined by NMR analysis, in agreement with previous results.^34^ Both functionalized polymers were obtained by derivatization of part of the lactic acid side groups of **P100** with either ETSA (**P100E15**) or Boc-Cys(Trt)-OH, which was followed by TFA deprotection of the latter to yield **P100C15**. The extents of ETSA and Boc-Cys(Trt)-OH derivatization of available lactate side chains were 14.0 and 12.1 mol % units per polymer chain (feed was 15 mol % in both cases, indicating high derivatization yields) with *M*_n_ values of 23 and 17 kDa, respectively, as determined by NMR analysis. The higher *M*_n_ of **P100E15** is likely caused by the purification procedure using diethyl ether, with lower-molecular-weight polymer chains failing to precipitate. The ETSA modification results in a more hydrophobic polymer (and therefore has a higher solubility in the ether layer) than cysteine, which explains why an increased *M*_n_ was not observed for **P100C15**. GPC analysis showed that the molecular weight and molecular weight distribution of both modified polymers were well conserved (see Figure S2.1). The CPs (above this temperature, micelles are formed) were 33, 5, and 18 °C for **P100**, **P100E15**, and **P100C15**, respectively. Derivatization with more hydrophobic handles explains the observed decrease in CP, with ETSA being a more hydrophobic functionalization than cysteine as also observed previously.^19^

### CCPM Synthesis

**Figure 1:**
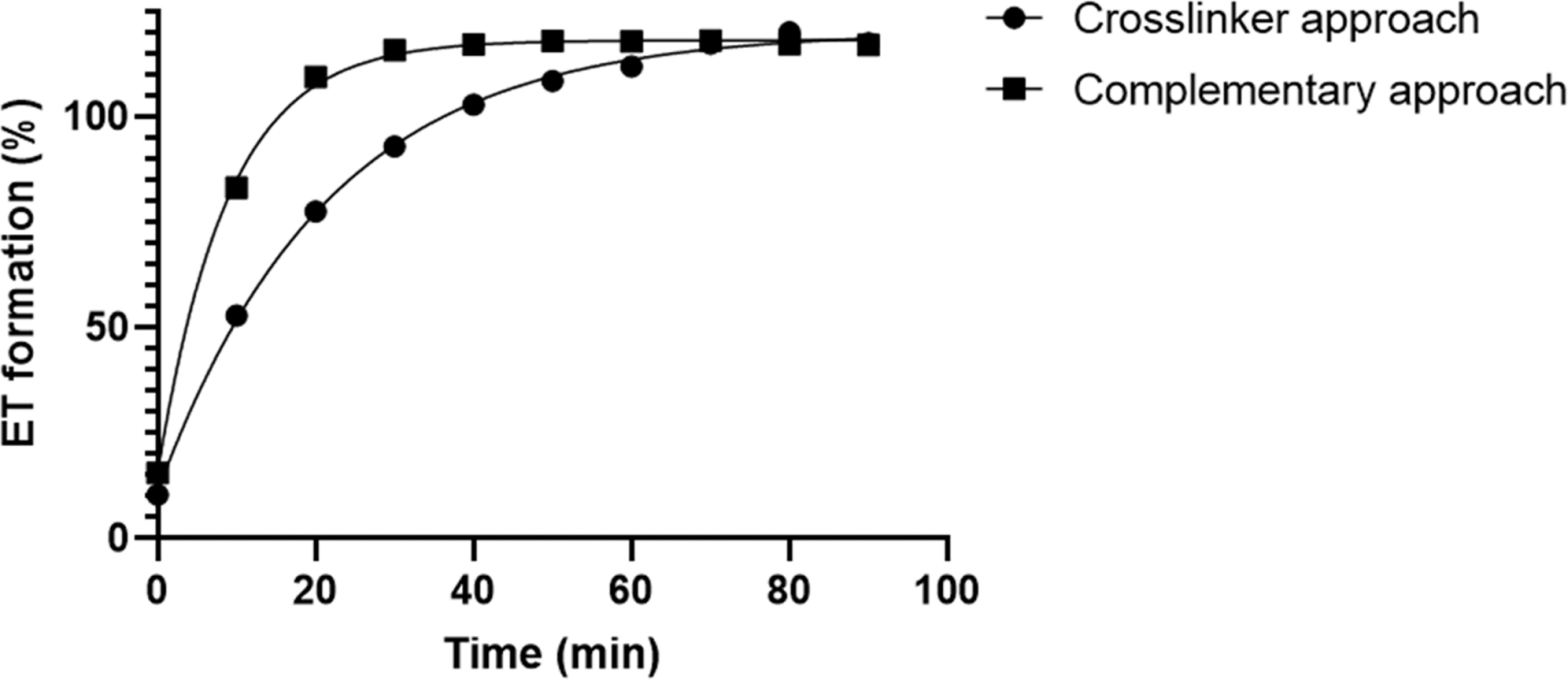
Kinetics of ethyl thioglycolate (ET) formation from the NCL crosslinking reaction via the crosslinker (circles) and complementary polymer (squares) approach, determined by UHPLC and expressed as a percentage of the maximal ET that can be formed theoretically.

CCPMs were formed by using either **P100E15** with the crosslinker (compound **2**) or **P100E15** with **P100C15**, with ETSA in slight excess compared to cysteine residues. The formation of ethyl thioglycolate (a byproduct of the NCL reaction) was determined by UHPLC (Figure 1) and reflects the extent of amide bonds and thus crosslink formation.

Figure 1 shows that ET was formed as expected with approximately 115% ET formation in both crosslinking systems after 90 min (expressed as a percentage of ET release expected based on the cysteine feed). The 115% ET formation falls in the range of experimental errors expected from NMR measurements of the polymer(s) (±10%), ET concentration determination (±2–5%), and weighing (±1–2%). Importantly, the cysteine groups had quantitatively reacted within 90 min. The concentration of **P100E15** in the crosslinker approach is twice as high as in the complementary polymer approach and approximately the same holds for the cysteine content (exemplified in Scheme 5); therefore, there is a similar crosslink density (defined as the total of both inter- and intramolecular links) in both systems. The initial slower rate of ET formation in the crosslinker approach may be explained by the hydrophilic crosslinker having to diffuse into the hydrophobic micellar core, in contrast to the complementary approach where reactive groups are in close proximity upon micellization.

TFF purification was done to remove the formed ET and remaining impurities (such as TCEP). Polymer losses due to purification were investigated by total hydrolysis and subsequent lactic acid quantification by UHPLC, similar to a previously published procedure.^34^ Losses typically ranged between 20 and 30%, resulting in a total polymer concentration of approximately 15 mg/mL, out of the initial 20 mg/mL feed during crosslinking.

**Figure 2:**
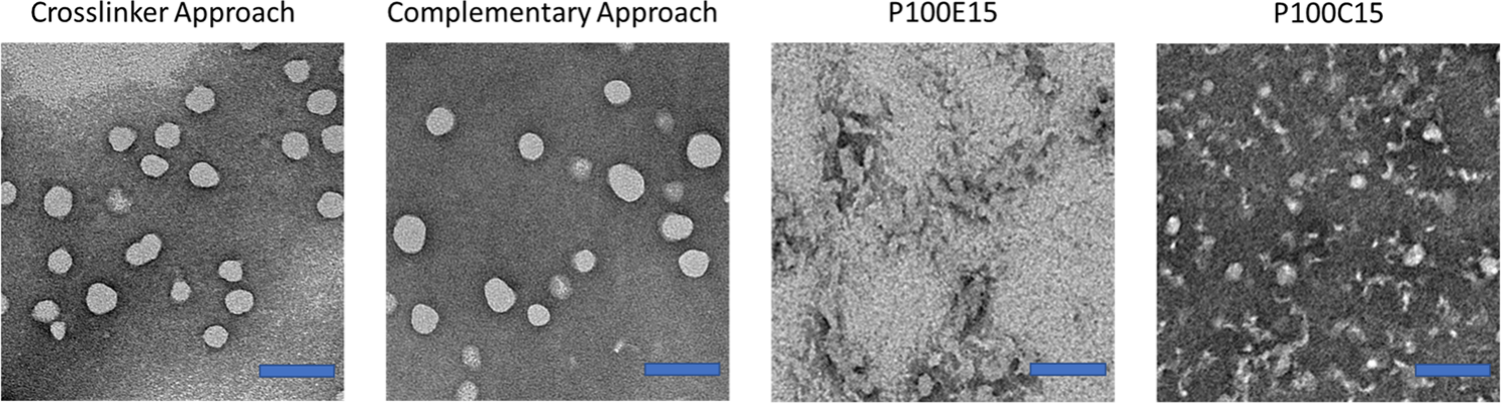
Negative staining TEM images of the crosslinker and complementary polymer-based CCPMs after purification as well as non-crosslinked polymeric micelles based on **P100E15** and **P100C15**. Scale bars are set to 100 nm.

TEM imaging confirmed that crosslinking by either approach resulted in stable spherical particles in the 50 nm diameter size range, in contrast to non-crosslinked free polymer samples that destabilize during preparation with the negative staining technique (see Figure 2).

### CCPM Degradation

The degradation of the two different CCPMs was studied under physiological conditions (pH 7.4, 37 °C).

#### Batch-Mode DLS Measurements

**Figure 3:**
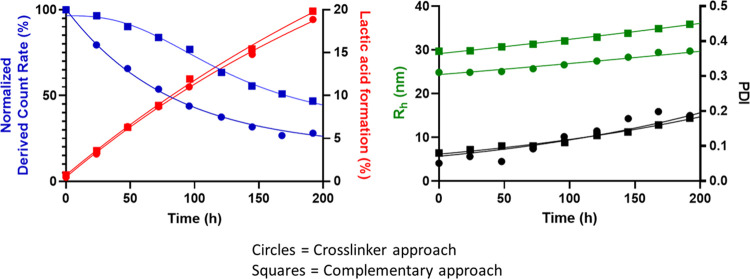
Degradation characteristics of crosslinker (circles) and complementary (squares) polymer based CCPMs under physiological conditions (pH 7.4, 37 °C) at a polymer concentration of ∼15 mg/mL. Normalized derived count rate (blue), *R*_h_ (green), and PDI (black) were determined by DLS measurements at 37 °C. Lactic acid formed % (red) was determined by UHPLC.

Figure 3 shows the degradation of the two different CCPMs as determined by batch-mode DLS (direct bulk measurement of a sample in a cuvette). The scattering intensity (SI, a metric previously employed to follow CCPM degradation^10,34^) depends on the particle concentration, particle radius, and refractive index increment of the CCPMs.^40^ However, for a dispersion with a fixed particle concentration and particle mass, SI increase in particle size due to swelling is exactly compensated for by the resulting change in the specific refractive index increment (see Supporting Information Section 5).^41−43^ Interestingly, Figure 3 shows an immediate decrease in SI in time for the crosslinker-based CCPMs as we also observed previously,^34^ whereas the SI of complementary polymer CCPMs showed a decrease after a certain lag time of 25–50 h. The time to decrease SI to 50% was also substantially different, approximately 80 h and 170 h for the crosslinker and the complementary polymer approach, respectively. The percentage amount of lactic acid formation was equal for both systems, ruling out different hydrolysis rates (possibly resulting from different core environments) being responsible for the observed difference in the decrease of SI. The mass loss due to lactic acid formation from both CCPMs after 192 h was approximately 9%, which cannot solely explain the observed decrease in SI. Furthermore, the lactic acid formation kinetics are in good agreement with previous kinetic data for a similar polymer.^44^

Shortly after preparation, the crosslinker CCPMs based on **P100E15** and compound **2** had a smaller radius of hydration (*R*_h_) than the complementary CCPMs based on **P100E15** and **P100C15** (Table 2), which can be explained by the higher hydrophobicity of **P100E15** (as compared to **P100C15**) that results in micelles with a lower extent of hydration of the hydrophobic core of the micelles. During the 192 h incubation at pH 7.4 and 37 °C, the *R*_h_ of the CCPMs increased to 28 and 35 nm for the crosslinker and complementary approach, respectively, which is discussed in the Degradation Mechanism section. The polydispersity index (PDI) increased during degradation, which is also in line with previously published findings, being associated with the degradation of particles in batch DLS measurements.^10,34^ Similar results were obtained by accelerated degradation of the CCPMs at pH 9.5, 25 °C (Figure S4.1). The accelerated degradation is due to the increased rate of lactate ester hydrolysis, which is driven by first-order kinetics with respect to OH^–^ concentration above a pH of 5.^8,45^ Taken together, it is demonstrated that CCPMs prepared by reacting **P100E15** with **P100C15** (complementary approach) degrade slower than CCPMs obtained by reaction of **P100E15** with compound **2** (crosslinking approach).

**Table 2:** Initial Micelle and CCPM Sizes as Determined by Batch DLS at 25 °C

	P100E15 not crosslinked	P100C15 not crosslinked	crosslinker approach	complementary approach
*R*_h_ (nm)	25	32	25	30

#### AF4/DLS/MALS Measurements

**Figure 4:**
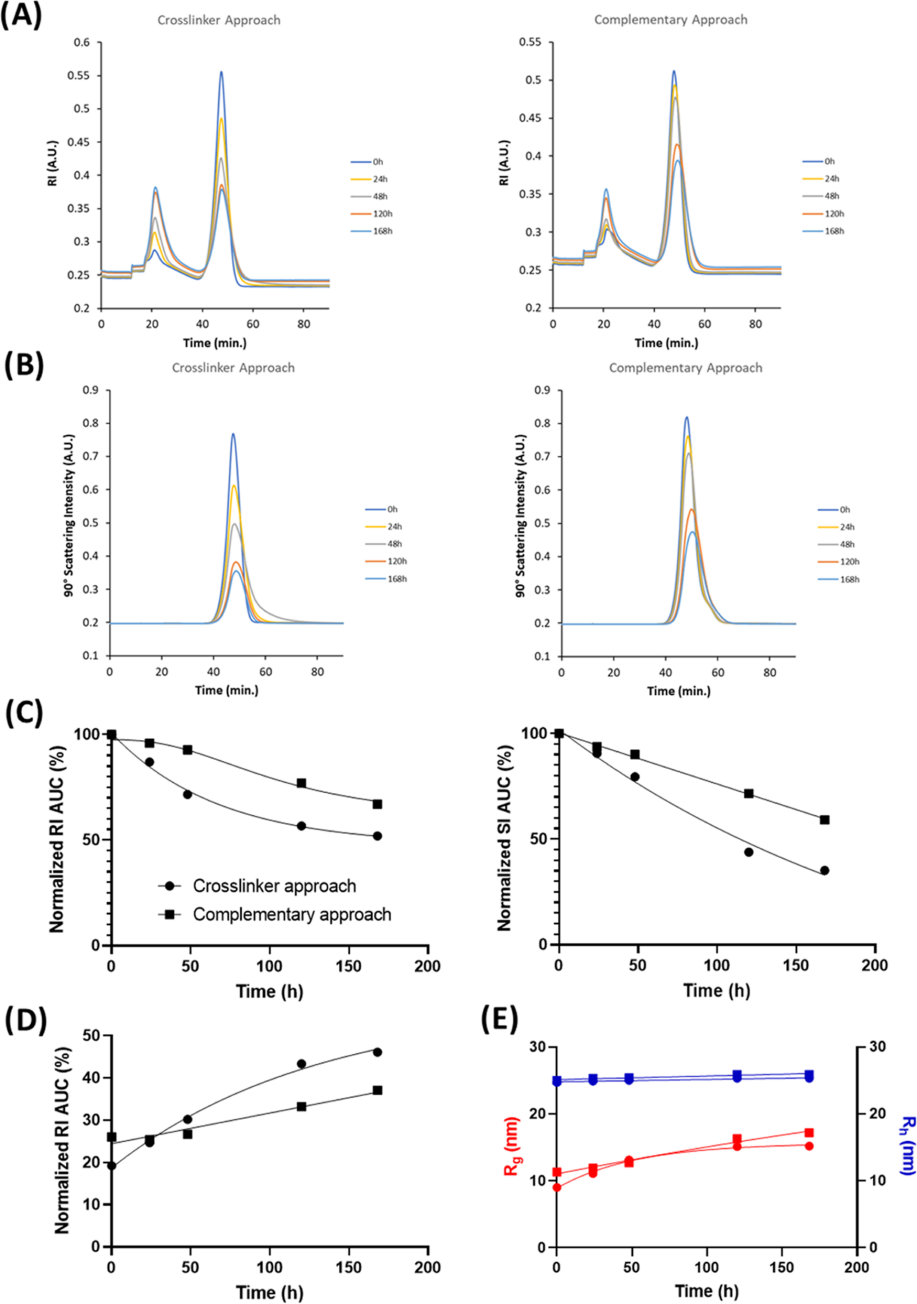
Degradation characteristics of crosslinker and complementary polymer-based CCPMs under physiological conditions (pH 7.4, 37 °C) at a polymer concentration of ∼20 mg/mL fractionated by AF4. (A) Fractograms recorded by the refractive index, (B) fractograms recorded by SLS at a 90 °C angle, (C) normalized refractive index and scattering intensity AUC of the CCPM signals (*R*_t_ = 50 min) in fractograms of (A) and (B), respectively, (D) normalized refractive index AUC of the *R*_t_ = 22 min peaks in the fractograms of (A) and (E) peak SI *R*_g_ and *R*_h_ values recorded by MALS and DLS (circles, crosslinker approach; squares, complementary polymer approach). Peaks at *R*_t_ = 50 min represent CCPMs, and peaks at *R*_t_ = 22 min represent soluble (partially) hydrolyzed mPEG-*b*-P(HPMAmLac*_n_*), P(HPMAmLac*_n_*), P(HPMA), and mPEG polymers, which are chemically not connected to the CCPMs.

The degradation of CCPMs was also studied using AF4 to analyze the CCPM distribution and soluble polymer species formed during hydrolysis. A decrease in both RI and 90° light scattering signal resulting from eluting CCPMs (*R*_t_ = 50 min) over the degradation time points was observed (Figure 4A,B), with a faster decrease for the CCPMs prepared using the crosslinker approach. The RI fractograms (Figure 4A) also show an increase of soluble polymer species (Figure 4D), smaller in size as hallmarked by their short retention time (*R*_t_ = 22 min) and lack of light scattering (Figure 4B). Given that a 10 kDa membrane was employed for the fractionation, these emerging peaks are likely soluble mPEG-*b*-P(HPMAmLac*_n_*) chains, which are partially hydrolyzed with a CP above 25 °C (AF4 measurement temperature) and no longer chemically linked to the CCPMs. Additionally, partially hydrolyzed P(HPMAmLac*_n_*) as well as fully hydrolyzed P(HPMA) and mPEG polymers can be expected following the ester bond cleavage connecting the two blocks.^37^ The values of the integrated CCPM RI peaks in time are shown in Figure 4C and support the trend also found by batch DLS in Figure 3, namely, a faster degradation of the crosslinker-based CCPMs as compared to the complementary polymer CCPMs. Importantly, the SI intensity decrease shown in Figure 4C is comparable to the SI measured by batch DLS in Figure 3. In Figure 4E, values of *R*_g_ and *R*_h_ at the peak SI are reported (in contrast to batch DLS where the entire sample was measured). Surprisingly, Figure 4E shows that the radius of hydration (*R*_h_) at peak SI remained rather constant and equal for both CCPM types (25 nm). This contrast to Figure 3 is explained by the difference between batch DLS measurements (affected by the whole distribution) to the AF4 separation method, where the *R*_h_ at the highest SI (most abundant species in the distribution) is reported, highlighting the importance of size separation techniques for CCPM analysis.^46^ Furthermore, the radius of gyration (*R*_g_) at peak SI increased during degradation of both CCPM types (from 10 to 18 nm, meaning an increase in the *R*_g_/*R*_h_ ratio from 0.4 to 0.7), which suggests a loss in mass density due to hydrophilization of the core^47^ as can be expected from the hydrolysis of the lactic acid side chains.

### Degradation Mechanism

**Scheme 6:**
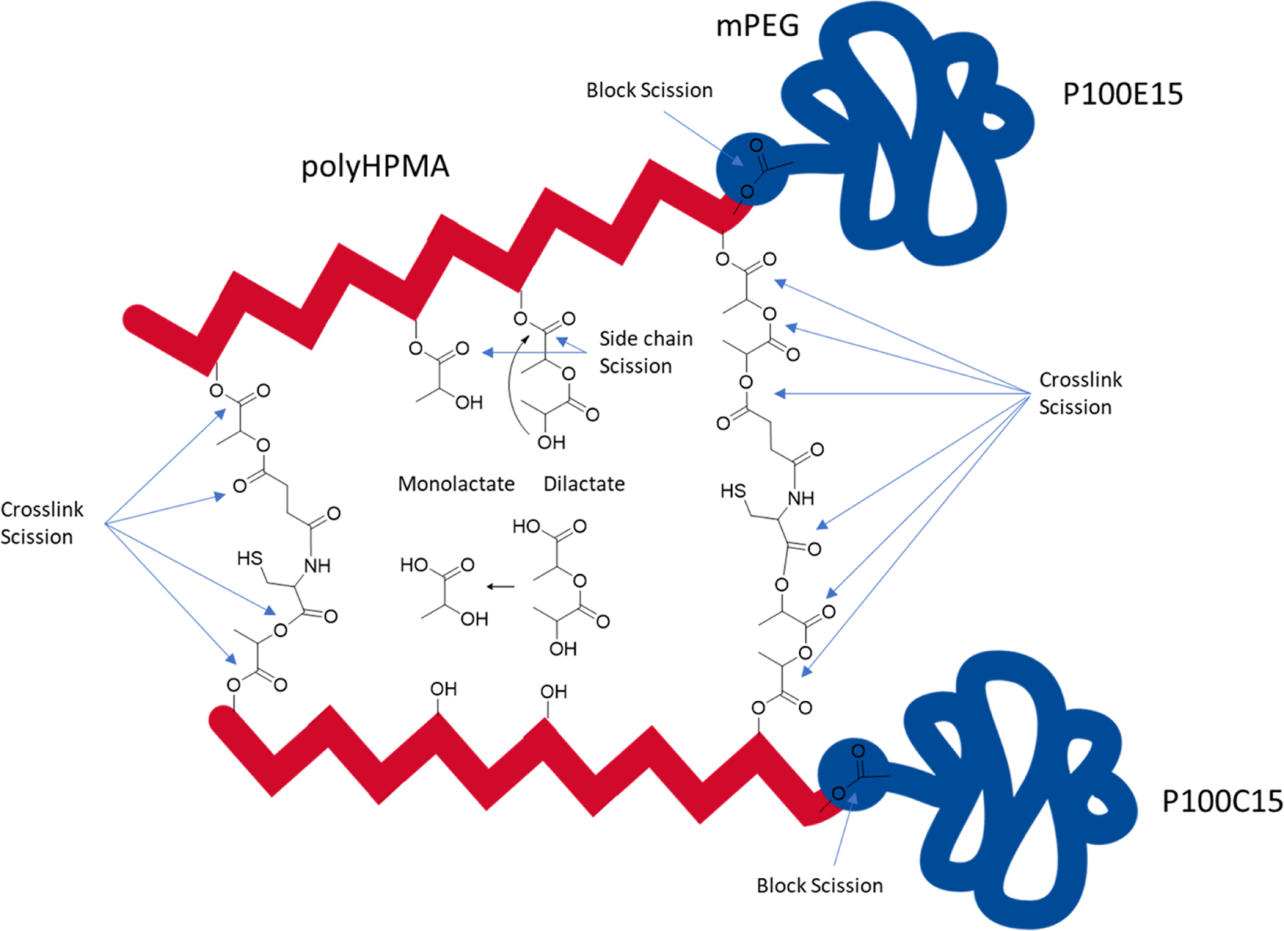
Hydrolysis Mechanism of NCL-Based CCPMs, Using the Complementary **P100E15** with **P100C15** CCPMs The dilactate side chains hydrolyze 6 times faster than both monolactate side chains and the esters involved in the crosslinks (4–6 esters per crosslink) due to an internal attack of the terminal alcohol.^8,37^ It is thus expected that dilactate side chains and crosslinks are cleaved at a similar rate (a crosslink requires only one scission to become ineffective).

Taken together, degradation of the CCPMs is due to hydrolysis of ester bonds present in HPMAmLac_1_ and HPMAmLac_2_ side chains and of the ester bonds present in crosslinks (Scheme 6). Further, an ester bond connects the mPEG block to the poly(HPMA) block, whose hydrolysis results in block scission.^37^ The hydrolysis rate of HPMAmLac_2_ is 6 times faster than that of HPMAmLac_1_ and is ascribed to the intramolecular attack of the terminal alcohol onto the carbonyl group connecting HPMA with the dilactate.^37^ HPMAmLac_2_ is a more hydrophobic monomer than HPMAmLac_1_ and is responsible for lowering the CP of HPMA-lactate-based polymers below 37 °C as has been shown previously.^30^ In fact, the CPs of PHPMAmLac_1_ and PHPMAmLac_2_ homopolymers have been reported to be 13 and 65 °C, respectively, indicating that hydrolysis of HPMAmLac_2_ units to hydrophilic HPMA units increases the CP above 37 °C. Thus, upon incubation of CCPMs at pH 7.4 and 37 °C, HPMAmLac_2_ side chains are hydrolyzed in time, resulting in a loss of mass as well as an increase in hydrophilicity of the core. The increase in hydrophilicity was also observed by UHPLC, where a decrease in retention time during degradation of the CCPM peaks was observed (see Figure S3.1 for chromatograms and Figure S4.3 for normalized AUCs). This increased hydrophilicity and resulting swelling of the core explain the observed slight increase in the hydrodynamic radius (*R*_h_) of the CCPMs measured by batch-mode DLS (Figure 3). Interestingly, this increase in size was not observed with the AF4 measurements of the CCPM species where the peak SI *R*_h_ remained constant but the peak SI *R*_g_ increased (Figure 4E). This finding highlights the more detailed distribution insights that separation techniques such as AF4 can provide in extension to batch-mode DLS, as has previously also been described.^46,48^ Nonetheless, batch-mode DLS provides the global average and in this work remains leading for the trends observed. Besides hydrolysis of lactic acid side chains, ester bonds in the crosslinks (4–6 esters are present per crosslink; see Scheme 6) are hydrolyzed, which finally results in formation of soluble HPMA-rich polymer chains that are extracted from the CCPMs. This loss of mass due to formation of lactic acid and soluble polymer chains is likely responsible for the observed decrease in scattering intensity in Figure 3 as well as Figure 4C. The increase in hydrophilicity as well as the loss of core mass due to HPMAmLac_2_ hydrolysis may explain the increase in *R*_g_ as shown in Figure 4C. Simultaneously, when the ester bonds in the crosslinks between the polymer chains are hydrolyzed, destabilization of the CCPMs occurs.

Assuming that a particle is degraded when the number of hydrolyzed crosslinks surpasses a critical value and that the hydrolytic rate constant for all crosslinks is equal, we simulated the degradation curves with regard to crosslink density in Figure S4.2, which shows that CCPMs with a low crosslink density undergo rapid decay, whereas higher crosslink densities result in an S-curve with a lag time before decaying.

Moving back to our hypothesis on how the crosslinker or complementary CCPM formation approach affects the degradation characteristics, the complementary approach clearly showed slower degradation in terms of SI and RI signal while still showing a similar swelling behavior (*R*_h_ and *R*_g_) and lactic acid formation as the crosslinker approach (hydrolysis proceeds equally fast). Since a comparable amount of crosslinks (intermolecular and potential intramolecular) was formed as discussed in the CCPM Synthesis section, this strongly suggests that the complementary approach resulted in CCPMs with a significantly higher effective (intermolecular) crosslink density than the crosslinker approach. This is best explained by intrachain reactions of the crosslinker with the same polymer, lowering the effective crosslink density.

## Conclusions

CCPMs were formed by two different crosslinking strategies: with a bifunctional crosslinking agent or by using a complementary polymer approach, employing native chemical ligation as a crosslinking reaction. We aimed to study and compare the degradation of these two types of CCPMs under physiological conditions. It was found that the crosslinker-based CCPMs had a considerably faster degradation rate as compared to the complementary polymer approach. This is likely due to the occurrence of intramolecular reactions resulting in ineffective crosslinks by using a bifunctional crosslinker, which are avoided by employing complementary polymers. These intramolecular crosslinking inefficiencies of a crosslinker approach certainly also apply in a broader scope to other CCPM systems and should be taken into consideration in their design.

## Supporting Information Available

^1^H NMR spectra of the (de)protected crosslinker, **P100**, **P100E15**, and protected **P100C15** (Figures S1.1–S1.5), ^19^F NMR spectrum of the deprotected crosslinker (Figure S1.6), GPC chromatograms of **P100**, **P100E15**, and deprotected **P100C15** (Figure S2.1) and UHPLC chromatograms of different degradation time points of the CCPMs formed via the crosslinker or the complementary polymer approach (Figure S3.1), CCPM degradation characteristics measured by DLS under accelerated conditions (pH 9.5, 25 °C) (Figure S4.1), simulation of expected particle degradation with varying crosslinking densities (Figure S4.2), and a mathematical explanation of expected changes in the SI of swelling particles in Section 5 (Figure S4.3) (PDF)

## Abbreviations

ABCPA: 4,4-azobis(4-cyanopentanoic acid)
ACN: acetonitrile
AF4: asymmetric flow field flow fractionation
CCPM: core-crosslinked polymeric micelle
CMC: critical micellization concentration
CP: cloud point
CMT: critical micellization temperature
DCC: *N,N*′*-*dicyclohexylcarbodiimide
DCM: dichloromethane
DCR: derived count rate
DCU: dicyclohexylurea
DLS: dynamic light scattering
DMF: dimethyl formamide
DMSO: dimethyl sulfoxide
DPTS: *N,N*-dimethylaminopyridinium *p*-toluenesulfonate
ETSA: ethyl thioglycolate-succinic acid
GPC: gel permeation chromatography
HATU: hexafluorophosphate azabenzotriazole tetramethyl uronium
HPMAmLac_n_: *N*-2-hydroxypropyl methacrylamide mono-/dilactate
HPLC: high-pressure liquid chromatography
MALS: multiangle light scattering
mPEG: methoxy poly(ethylene glycol)
NCL: native chemical ligation
PDI: polydispersity index
PEG: poly(ethylene glycol)
PM: polymeric micelle
**P100**: mPEG_5000_-*b*-P(HPMAmLac_1_-*co*-HPMAmLac_2_) copolymer
**P100C15**: mPEG_5000_-*b*-P(HPMAmLac*_n_*-*co*-HPMAmLac*_n_*-cysteine) copolymer
**P100E15**: mPEG_5000_-*b*-P(HPMAmLac*_n_*-*co*-HPMAmLac*_n_*-ETSA) copolymer
PTFE: poly(tetrafluoroethylene)
RI: refractive index
SLS: static light scattering
TCEP: tris(2-carboxyethyl)phosphine
TEM: transmission electron microscopy
TFA: trifluoroacetic acid
UHPLC: ultra-high-performance liquid chromatography
