# Supplementary material for: Mechanistic Study on the Degradation of Hydrolysable Core-Crosslinked Polymeric Micelles
Source: Langmuir. 2023 Aug 15;39(34):12132–43. doi: 10.1021/acs.langmuir.3c01399 (PMC10469444; doi:10.1021/acs.langmuir.3c01399)
Supplement: Supplementary file 1 — la3c01399_si_001.pdf [file la3c01399_si_001.pdf]

Supporting Information (SI)

A mechanistic study on the degradation of hydrolysable core-crosslinked polymeric micelles

Erik R. Hebels[1], Mies J. van Steenbergen[1], Ragna Haegebaert[3], Cornelis W. Seinen[4], Barbara S. Mesquita[1], Antoinette van den Dikkenberg[1], Katrien Remaut[3], Cristianne J. F. Rijcken[2], Bas G.P. van Ravensteijn[1], Wim E. Hennink[1] and Tina Vermonden[1*]*

[1] Department of Pharmaceutics, Utrecht Institute for Pharmaceutical Sciences (UIPS), Utrecht University, 3508 TB Utrecht, the Netherlands.

[2] Cristal Therapeutics, 6229 EV Maastricht, the Netherlands

[3] Laboratory for General Biochemistry and Physical Pharmacy, Ghent University, Ottergemsesteenweg 460, 9000 Gent, Belgium

[4] Division Laboratories, Pharmacy and Biomedical Genetics, Central Diagnostic Lab, University Medical Center Utrecht, Heidelberglaan 100, 3584 CX Utrecht, the Netherlands

1. NMR Spectra

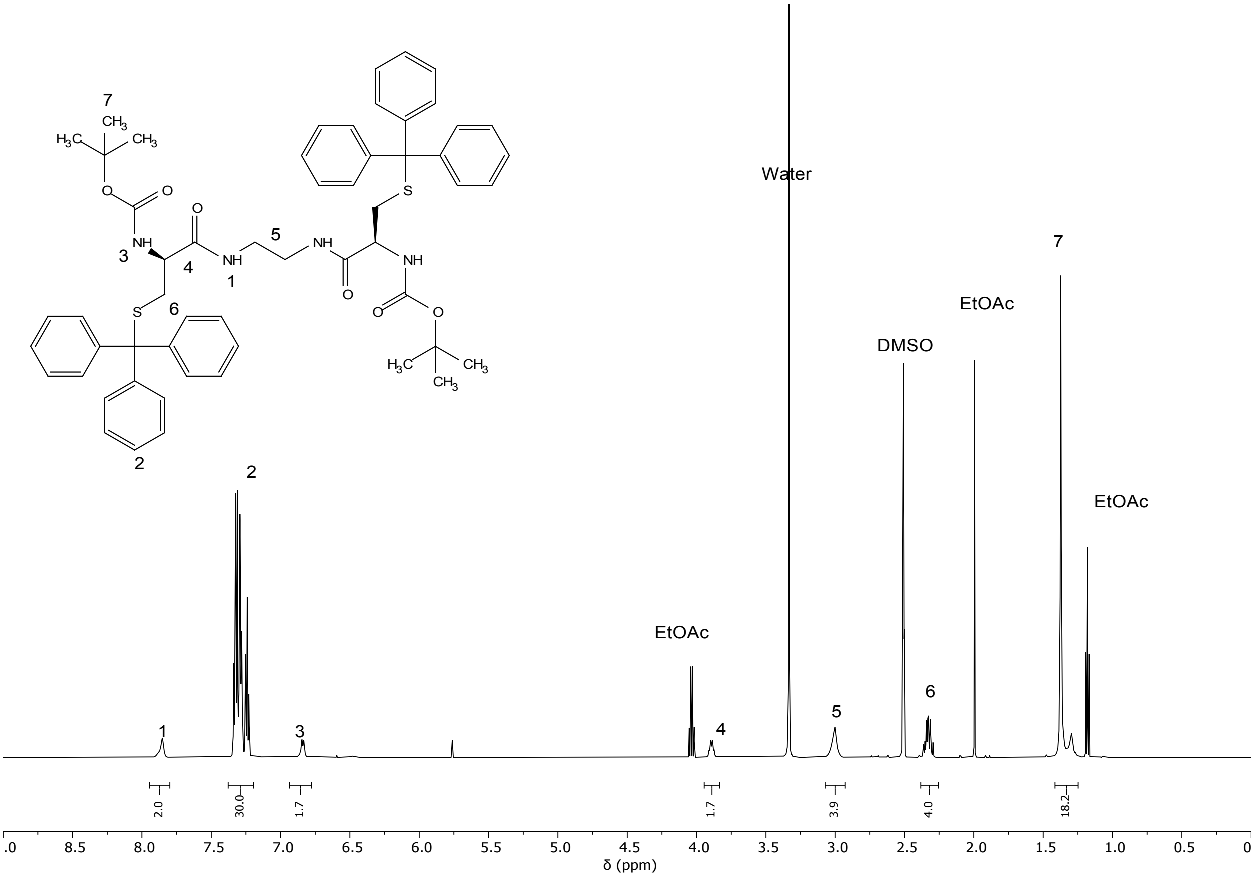

*Figure S1.1: ^{1}H NMR spectrum of the protected dicysteine crosslinker (compound **1**). The solvent was deuterated DMSO.*

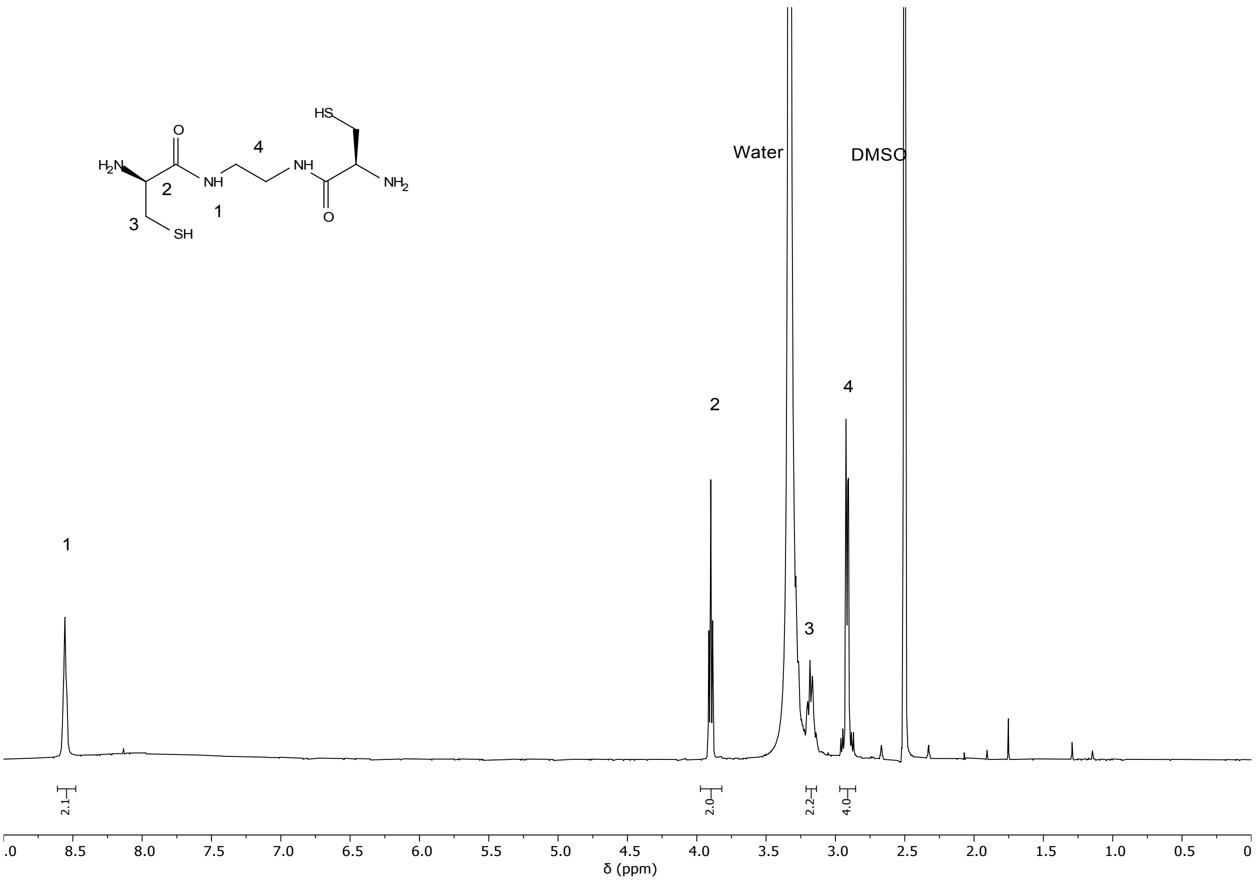

*Figure S1.2: ^{1}H NMR spectrum of the dicysteine crosslinker (compound **2**). The solvent was deuterated DMSO. Spectrum matches a previously reported one.* [1] *Although formic acid was employed as modifier during the Prep-RP-HPLC purification procedure, the formate counterion signal was not detected in the ^{1}H NMR spectrum. Through ^{19}F NMR (figure S1.6), it was shown that TF-acetate resulting from the deprotection employing TFA remained the exclusive counterion of the amine salt, which was hence incorporated into the molecular weight calculations of the crosslinker in this work. The challenges to exchange or remove TF-acetate salts in peptides have been described elsewhere.* [2]

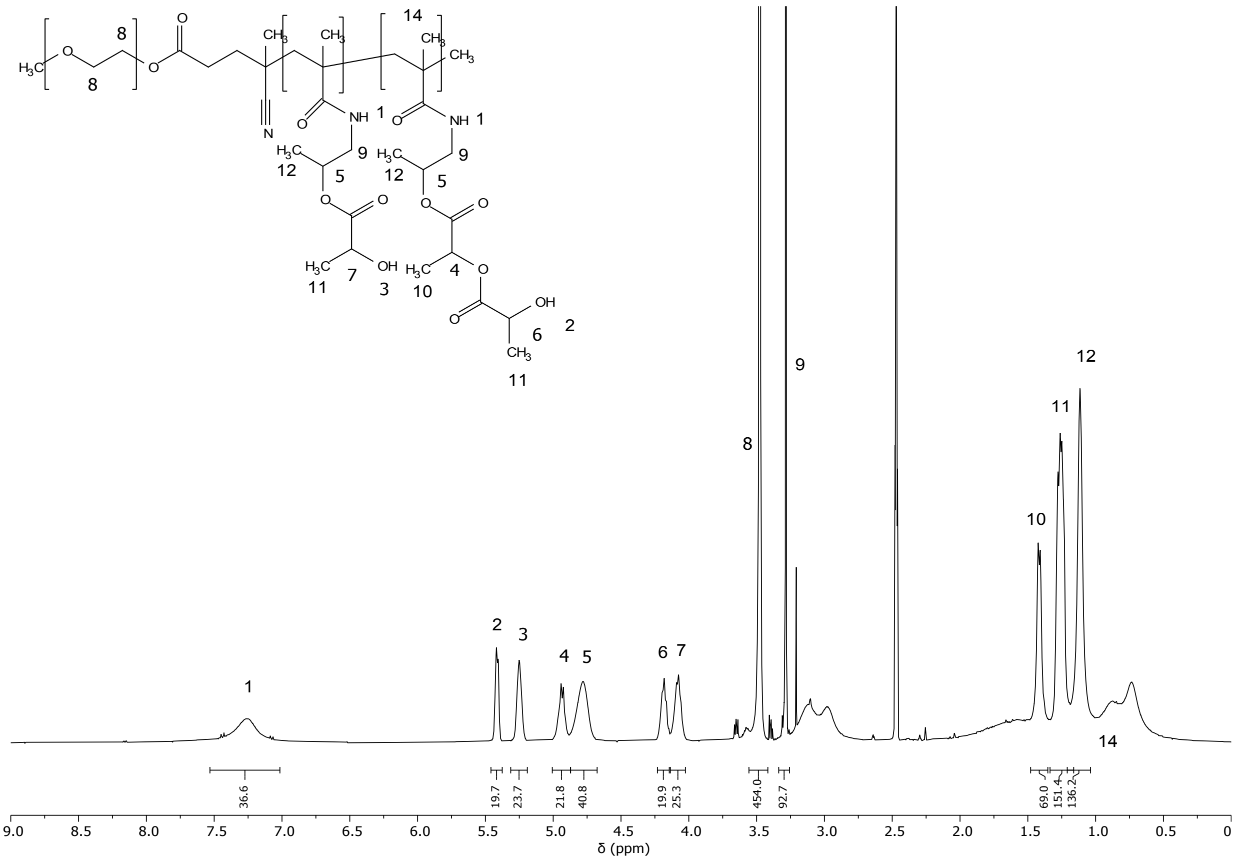

*Figure S1.3: ^{1}H NMR spectrum of **P100** (PEG$_{5000}$-b-P(HPMAmLac$_1$-co-HPMAmLac$_2$)). The solvent was deuterated DMSO.*

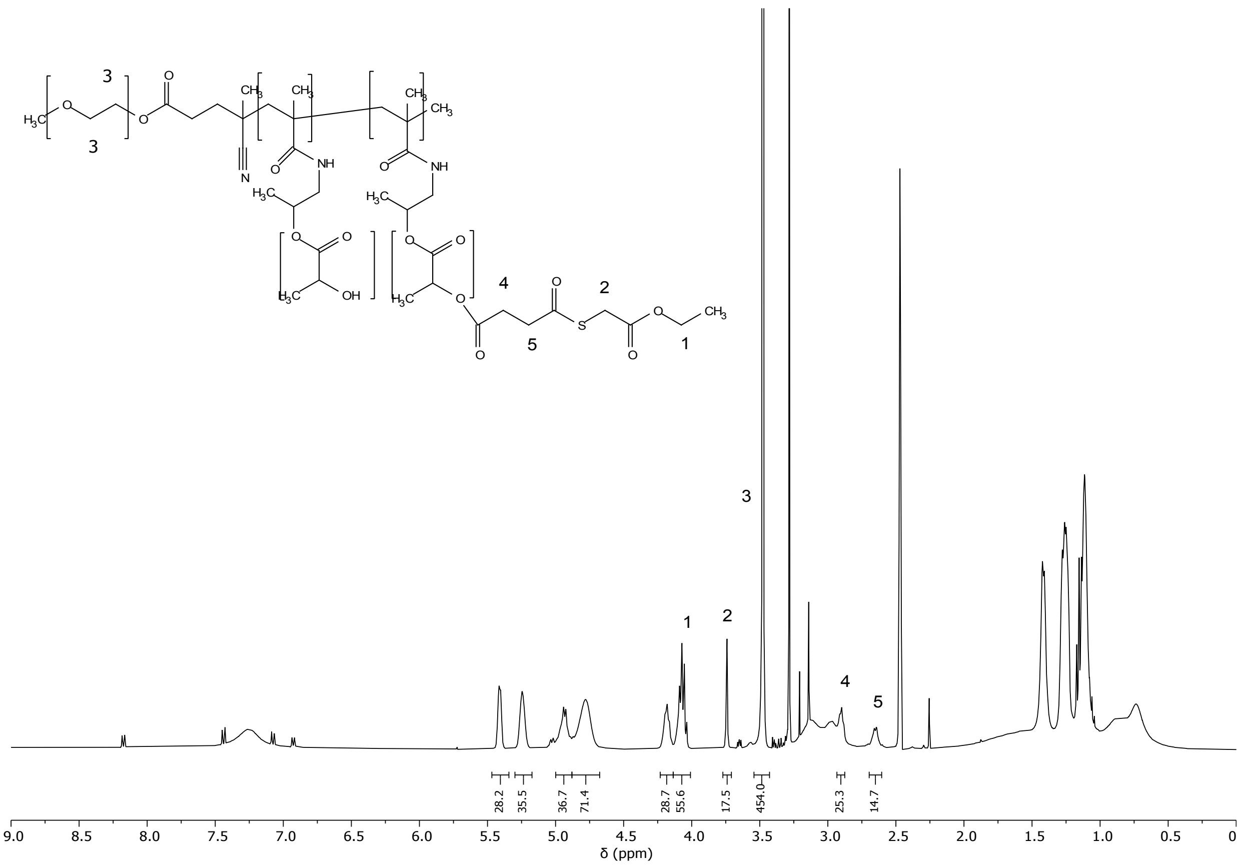

Figure S1.4: ^{1}H NMR spectrum of ***P100E15*** *(PEG$_{5000}$-b-P(HPMAmLac$_n$-co-HPMAmLac$_n$-ETSA)). The solvent was deuterated DMSO.*

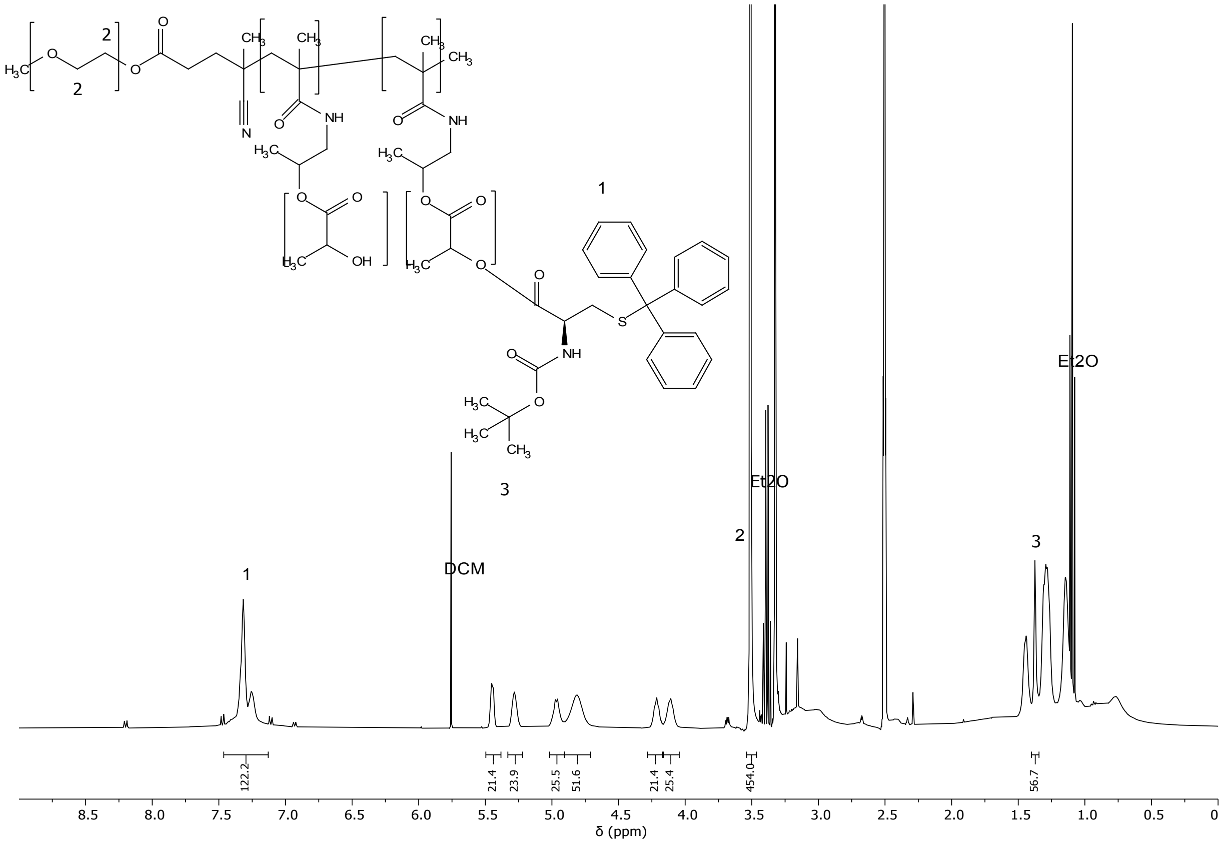

*Figure S1.5: ¹H NMR spectrum of **P100C15 prot** (PEG$_{5000}$-b-P(HPMAmLac$_n$-co-HPMAmLac$_n$-Cys(Trt)-Boc)). The solvent was deuterated DMSO.*

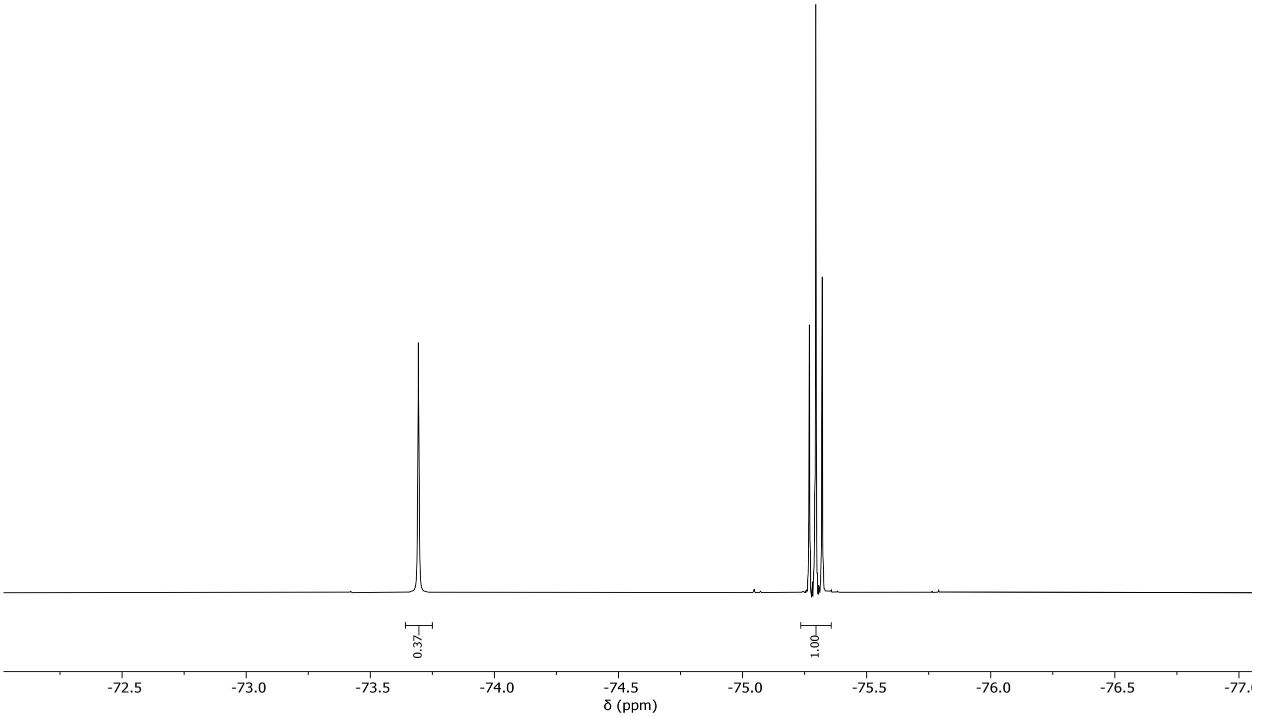

*Figure S1.6: ^{19}F NMR spectrum of the crosslinker (compound **2**, singlet signal) including an internal reference (trifluoroethanol, triplet signal). The amounts of crosslinker and trifluoroethanol were 6.0 mg (12 µmol) and 7.1 mg (71 µmol), respectively. The solvent was deuterated DMSO.*

2. GPC Chromatograms

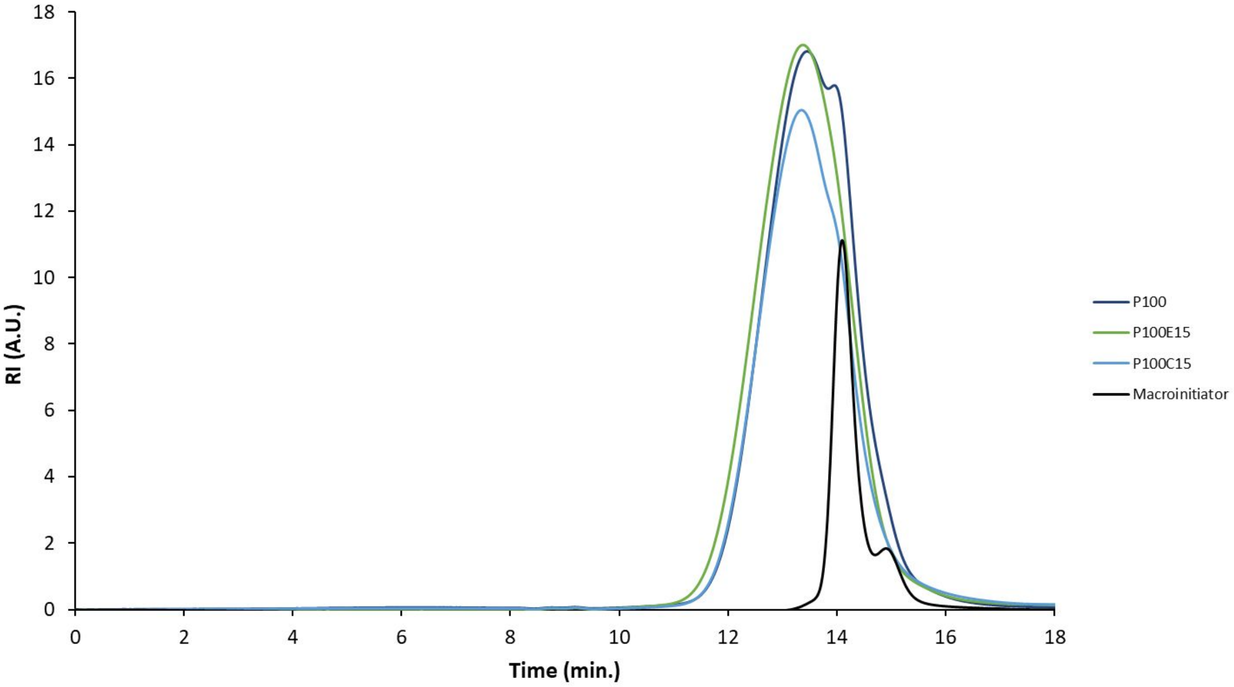

	GPC M_n (kDa)	Đ	NMR M_n (kDa)
**P100**	14.9	1.72	15.8 and 17.8
**P100E15**	17.0	1.80	22.5
**P100C15**	15.6	1.79	16.9
**Macroinitiator**	10.8	1.04	-

Figure S2.1: GPC chromatograms and analysis of ***P100*** *(blue) which was modified with either ETSA to* ***P100E15*** *(green) or Boc-Cys(Trt)-OH followed by deprotection to* ***P100C15*** *(light blue). Detection by RI. ETSA and Boc-Cys(Trt)-OH derivatization of available lactate side chains was 14.0 and 12.1 mol % units per polymer chain, respectively, as determined by NMR. The observed deviations in absolute values for Mn extracted from NMR and GPC are common, as both techniques rely on a different approach to extract these molecular weights. A small amount (<5%) of remaining macroinitiator (black) may be responsible for the slight shoulder observed in* ***P100****.*

3. UHPLC chromatograms

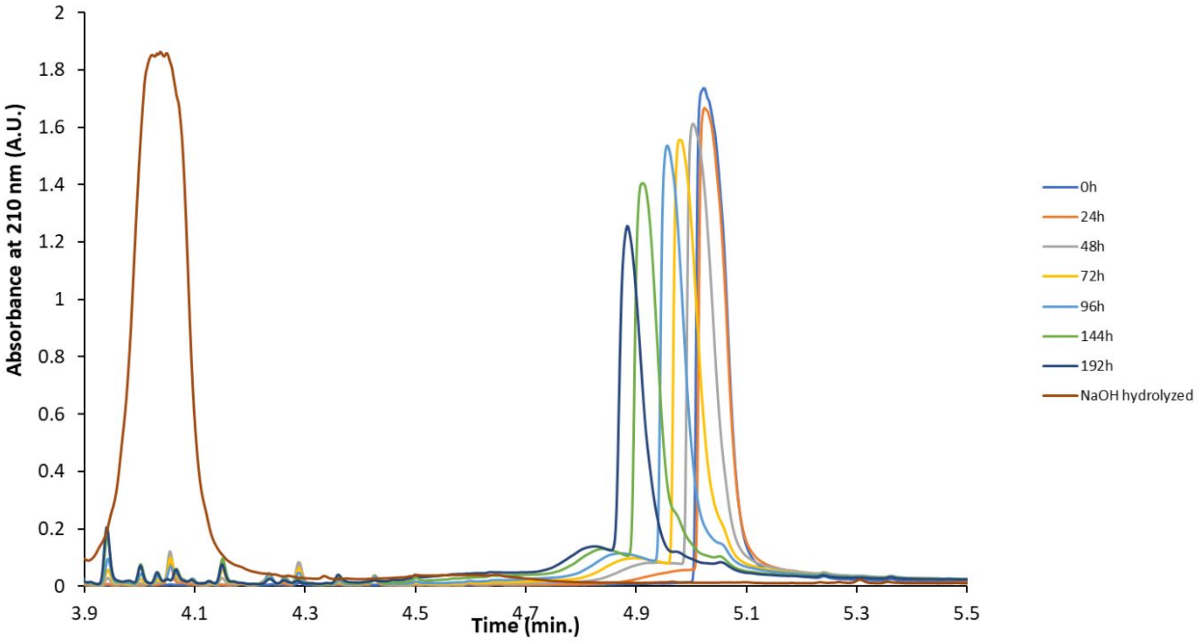

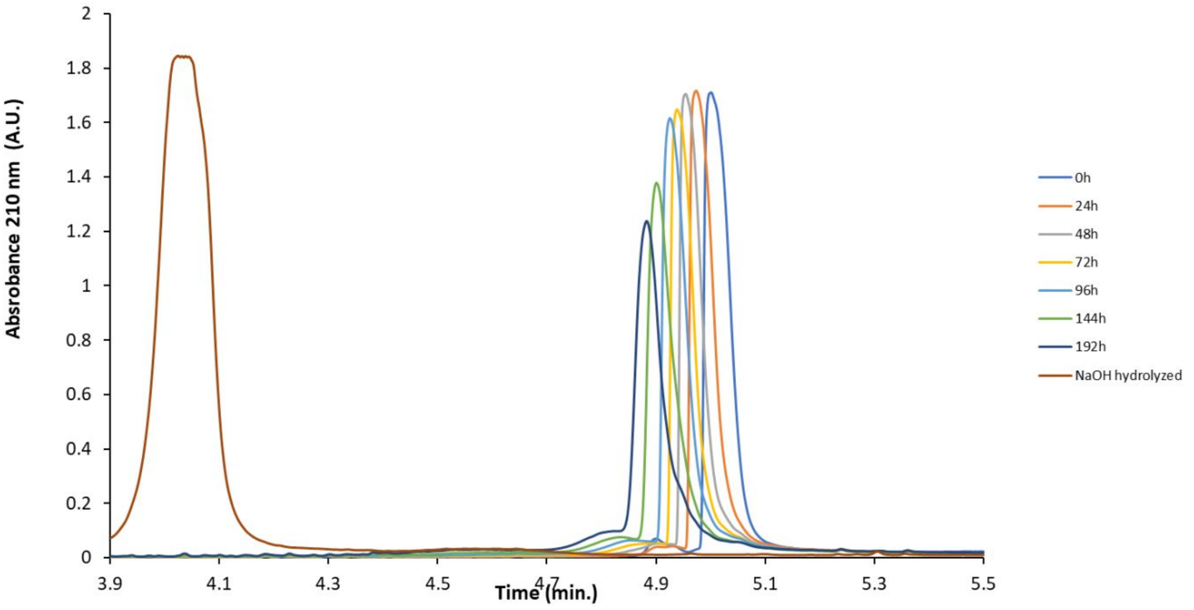

Figure S3.1: UHPLC chromatograms of degrading CCPMs (R_t between 4.8 and 5.1) formed by either the crosslinker (top) or complementary polymer (bottom) approach. Complete hydrolysis of the CCPMs resulted in the disappearance of the ascribed CCPM peaks and appearance of a more hydrophilic species at R_t = 4.0,

4. Additional figures

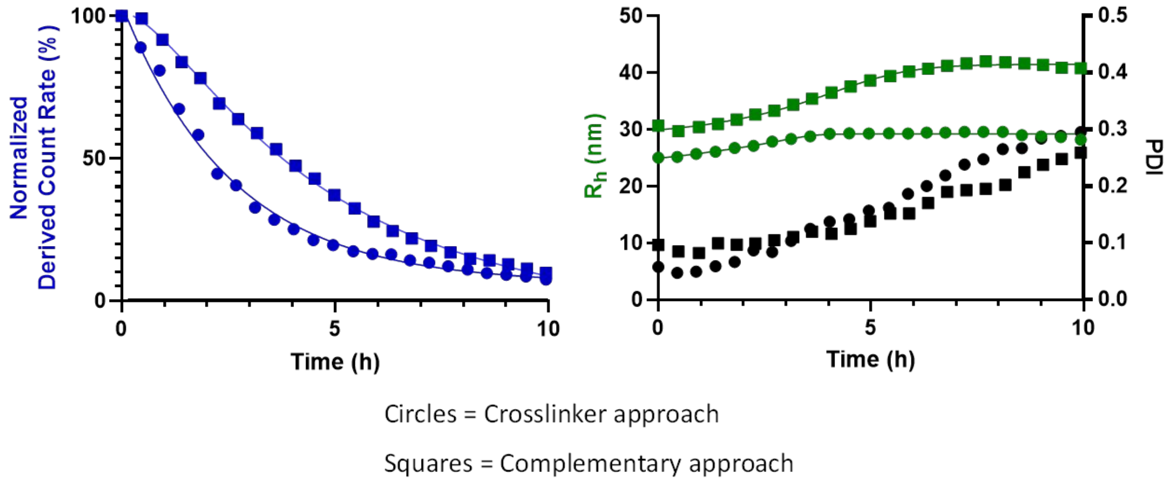

Figure S4.1: Degradation characteristics of crosslinker (circles) and complementary polymer (squares) based CCPMs under accelerated hydrolysis conditions (pH 9.5, 25 °C) at a polymer concentration of ~15 mg/mL. Normalized derived count rate (blue), Z-Ave (green) and PDI (black) were determined by DLS measurements at 25°C.

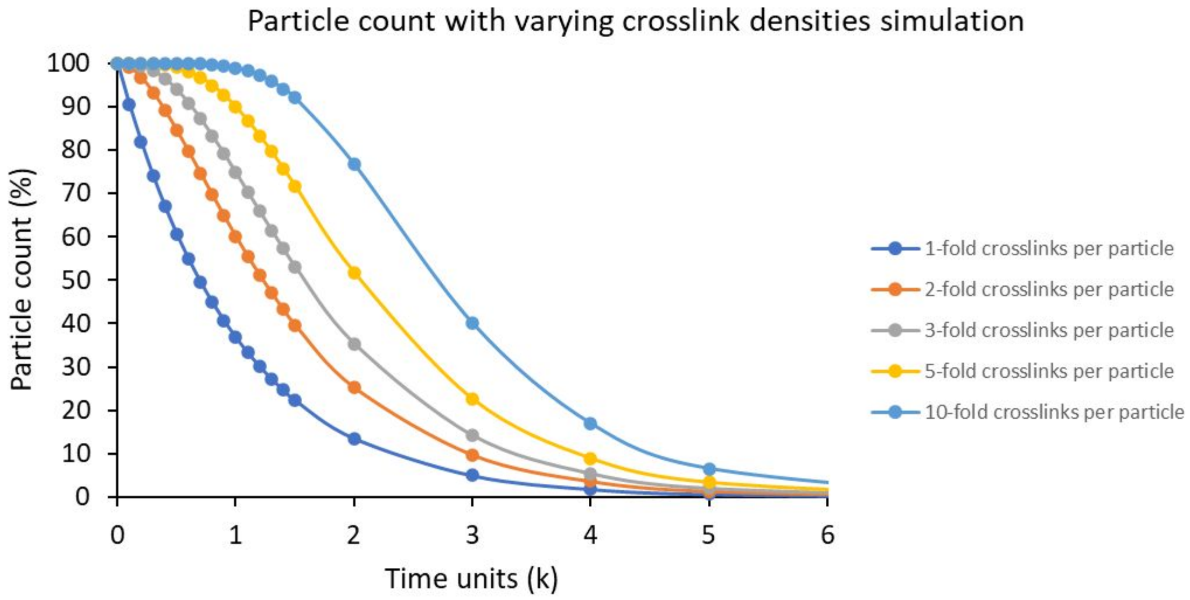

Figure S4.2: Simulated degradation characteristics of crosslinked particles undergoing hydrolysis with different crosslinking densities. The assumptions made are that a particle is degraded when all crosslinks are hydrolyzed (1-fold crosslinks being the critical number of crosslinks required for stability) and that the hydrolytic rate constant (following first order decay kinetics) for all crosslinks has the same value (taken as k = 1). With increasing percentage of crosslinks, a lag time for particle disintegration becomes apparent for the S-shaped degradation curves.

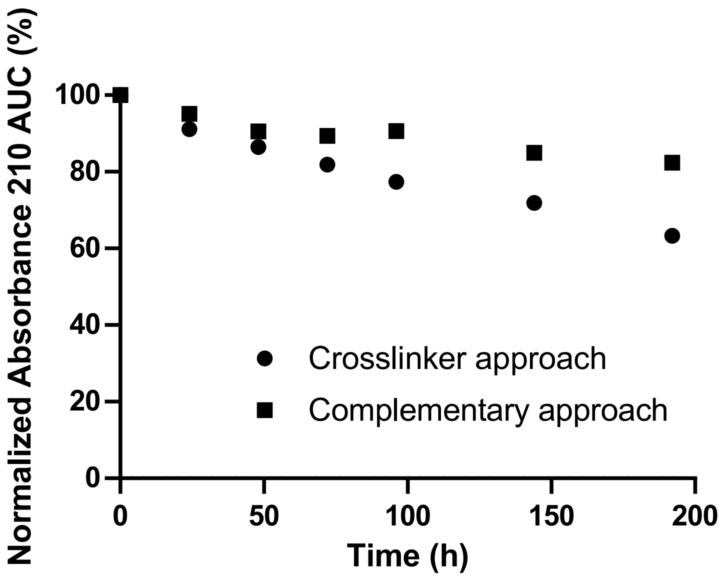

Figure S4.3: Degradation characteristics of crosslinker (circles) and complementary (squares) polymer based CCPMs under physiological conditions (pH 7.4, 37 °C), determined by UHPLC (R_t between 4.8 and 5.1) and recorded at 210 nm absorbance.

5. Swelling of colloidal particles and scattering intensity

Assuming that the particles are small compared to the wavelenght of light (λ), the scattered intensity (I_θ) at a certain angle (θ) can be described using the Rayleigh-Debye-Gans (RDG) formalism:

$$\frac{I_\theta}{I_0} \propto \lambda^{-4} \cdot V_p^2 \cdot \frac{m^2-1}{m^2+2} \cdot P(\theta) \quad \textbf{(1)}$$

where V_p is the volume of the RDG scatterer and *P(θ)* the particle's form factor. m is defined as the ratio of the refractive index of the particle (n_p) and the dispersing medium (n_s):

$$m = \frac{n_p}{n_s} \quad \textbf{(2)}$$

Since the V_p is proportional to the radius of the particle (R_p) to the power 6, Eq. **(1)** can also be written as, giving the well-known dependence of the scattered intensity to the particle size:

$$\frac{I_\theta}{I_0} \propto \lambda^{-4} \cdot R_p^6 \cdot \frac{m^2-1}{m^2+2} \cdot P(\theta) \quad \textbf{(3)}$$

Considering that $m \approx 1$ for most polymer/solvent combination, Eq. **(3)** can be simplified (via a Taylor expansion) to:

$$\frac{I_\theta}{I_0} \propto \lambda^{-4} \cdot R_p^6 \cdot (m-1)^2 \cdot P(\theta) \quad \textbf{(4)}$$

In the case of particles that swell by infusion of solvent, not only the dependence of the scattered intensity on the particle size, but also the change in n_p has to be considered when determining the scattered intensity. The refractive index of a composite particle (in this case polymer and solvent) can be written as:

$$n_p = \phi \cdot n_{polym} + (1 - \phi) \cdot n_s \quad \textbf{(5)}$$

with φ the volume fraction of polymer inside a single scatterer. This volume fraction is related to R_p:

$$\phi = \frac{V_{polym}}{V_{particle}} \propto R_p^{-3} \quad \textbf{(6)}$$

Rewriting $(m - 1)$ in terms of n_p, n_s and φ gives:

$$(m-1) = \left(\frac{n_p}{n_s} - 1\right) = \left(\frac{n_p - n_s}{n_s}\right) \quad \textbf{(7a)}$$

$$(m-1) = \frac{(\phi \cdot n_{polym} + (1-\phi) \cdot n_s) - n_s}{n_s} = \frac{\phi \cdot (n_{polym} - n_s)}{n_s} \quad \textbf{(7b}$$

Plugging this result into Eq. (4) yields:

$$\frac{I_\theta}{I_0} \propto \lambda^{-4} \cdot R_p^6 \cdot \phi^2 \left(\frac{n_{polym} - n_s}{n_s}\right)^2 \cdot P(\theta) \quad \textbf{(8)}$$

Considering the relation between φ and Rp (as given by Eq. **(6)**, the final relation for the scattered intensity of solven-swelling particles is obtained:

$$\frac{I_\theta}{I_0} \propto \lambda^{-4} \cdot R_p^6 \cdot R_p^{-6} \left(\frac{n_{polym} - n_s}{n_s}\right)^2 \cdot P(\theta) = \lambda^{-4} \left(\frac{n_{polym} - n_s}{n_s}\right)^2 \cdot P(\theta) \quad \textbf{(9)}$$

From Eq. **(9)** we learn that the scattered intensity becomes independent of the particle size, as increases in scattered intensity upon swelling are compensated by a loss of contrast between the dispersing medium and the particle core.

References

(1) Ziaco, B.; Pensato, S.; D'Andrea, L. D.; Benedetti, E.; Romanelli, A. Semisynthesis of Dimeric Proteins by Expressed Protein Ligation. *Org. Lett.* **2008**, *10* (10), 1955–1958.

(2) Roux, S.; Zékri, E.; Rousseau, B.; Paternostre, M.; Cintrat, J.-C.; Fay, N. Elimination and Exchange of Trifluoroacetate Counter-Ion from Cationic Peptides: A Critical Evaluation of Different Approaches. *J. Pept. Sci.* **2008**, *14* (3), 354–359.
